# MicroRNAs in Hepatocellular Carcinoma Pathogenesis: Insights into Mechanisms and Therapeutic Opportunities

**DOI:** 10.3390/ijms25179393

**Published:** 2024-08-29

**Authors:** Khadijeh Mahboobnia, Dianne J. Beveridge, George C. Yeoh, Tasnuva D. Kabir, Peter J. Leedman

**Affiliations:** 1 Laboratory for Cancer Medicine, Harry Perkins Institute of Medical Research, QEII Medical Centre, Perth, WA 6009, Australia; 22997944@student.uwa.edu.au (K.M.); dianne.beveridge@perkins.org.au (D.J.B.); george.yeoh@uwa.edu.au (G.C.Y.);; 2 Centre for Medical Research, The University of Western Australia, Perth, WA 6009, Australia; 3 School of Molecular Sciences, The University of Western Australia, Perth, WA 6009, Australia

**Keywords:** hepatocellular carcinoma, microRNAs, tumor heterogeneity, drug resistance, targeted therapy, delivery systems

## Abstract

Hepatocellular carcinoma (HCC) presents a significant global health burden, with alarming statistics revealing its rising incidence and high mortality rates. Despite advances in medical care, HCC treatment remains challenging due to late-stage diagnosis, limited effective therapeutic options, tumor heterogeneity, and drug resistance. MicroRNAs (miRNAs) have attracted substantial attention as key regulators of HCC pathogenesis. These small non-coding RNA molecules play pivotal roles in modulating gene expression, implicated in various cellular processes relevant to cancer development. Understanding the intricate network of miRNA-mediated molecular pathways in HCC is essential for unraveling the complex mechanisms underlying hepatocarcinogenesis and developing novel therapeutic approaches. This manuscript aims to provide a comprehensive review of recent experimental and clinical discoveries regarding the complex role of miRNAs in influencing the key hallmarks of HCC, as well as their promising clinical utility as potential therapeutic targets.

## 1. Introduction

### 1.1. Hepatocellular Carcinoma

Hepatocellular carcinoma (HCC) is a highly heterogeneous disease, accounting for 75–85% of human liver cancers, and is the third leading cause of cancer-related deaths worldwide [1,2]. Its incidence has been steadily increasing globally over the past three decades [1]. In Australia, there was a remarkable 378% increase in HCC cases between 1982 and 2015 [3]. The main causes of HCC are chronic infection with viral hepatitis (hepatitis B virus (HBV) and hepatitis C virus (HCV)), heavy alcohol consumption, and non-alcoholic fatty liver disease (NAFLD) [4]. As the prevalence of viral hepatitis has been reduced due to the global coverage of hepatitis B vaccination and the successful antiviral treatment for HCV, it is expected that NAFLD, which is associated with metabolic syndrome, type 2 diabetes, and central obesity, will be the major risk factor for HCC in the future [1,5,6]. These etiological factors disrupt crucial signaling cascades within hepatocytes, initiate tumor formation and heterogeneity, and compromise the subsequent treatment efficacy [7].

Three distinct transcriptomic signatures labelled S1, S2, and S3 have been identified in HCC, and their clinical severity being worse in S1 [8]. The S1 subtype is characterized by abnormal activation of the Wnt signaling pathway and overexpression of transforming growth factor beta 1 (TGFβ1), which correlates with an aggressive epithelial-to-mesenchymal (EMT) phenotype. S2 tumors have activated Myc and Akt pathways, and the upregulation of stemness markers such as α-fetoprotein (AFP) and epithelial cell adhesion molecule (EPCAM). In contrast, S3 tumors are smaller, more differentiated, and express genes associated with normal hepatocyte function, including glycolipid metabolism-associated genes and tumor suppressor genes p21 and p53, resulting in a less aggressive clinical profile [8].

Key pathways involved in HCC pathogenesis, including the receptor tyrosine kinase (RTK), fibroblast growth factor (FGF), PI3K/Akt and MAPK/ERK, WNT, Hedgehog, Notch, JAK/STAT, and ubiquitin-proteasome pathways, are dysregulated in liver cancer due to genetic and epigenetic factors [9]. Inflammatory hepatic lesions upregulate and activate various transcription factors and gene regulators that orchestrate these molecular cascades, driving different hallmarks of HCC, including uncontrolled cell growth, evasion of cell death signals, tumor microenvironment remodelling, immune escape, metabolic reprogramming, invasion, and metastasis [10].

### 1.2. microRNAs

**Figure 1:**
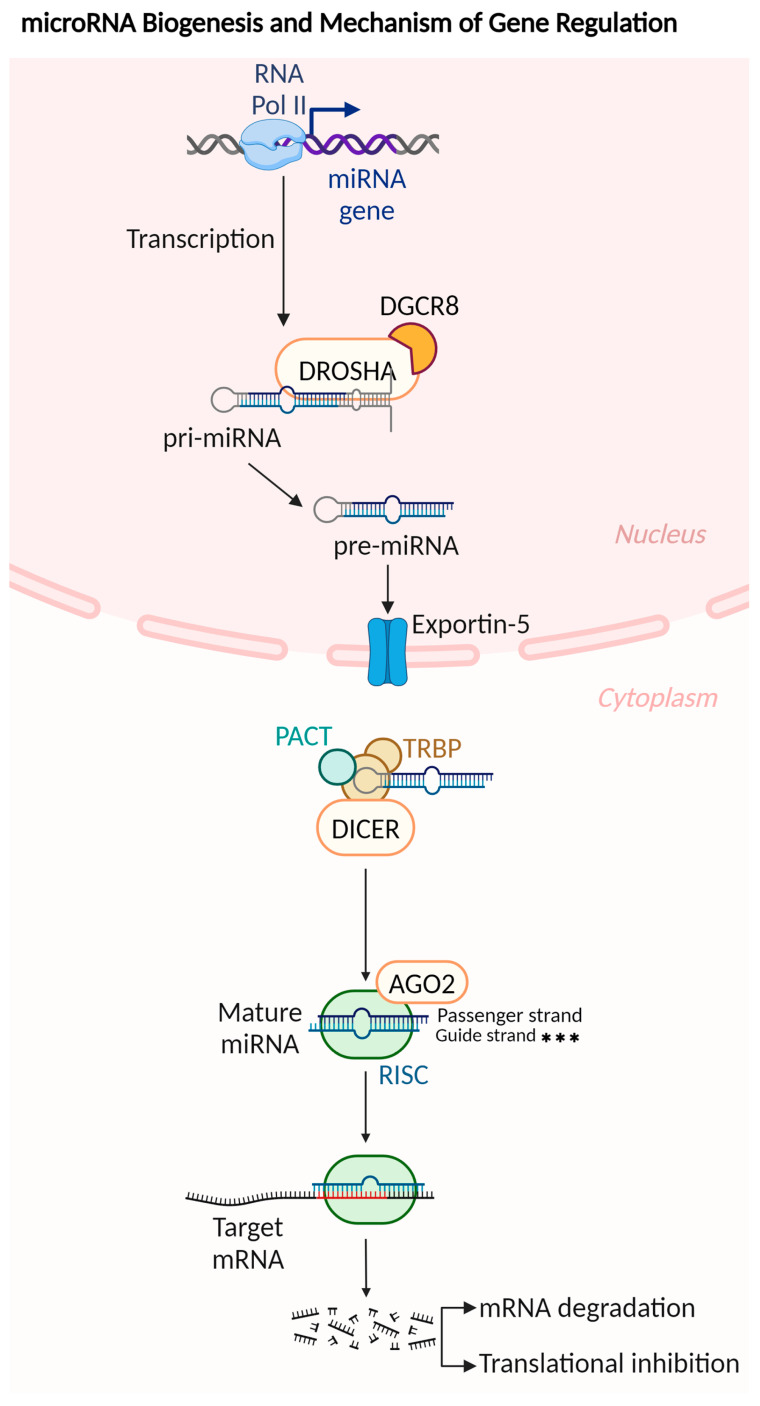
Schematic of miRNA biogenesis, illustrating the steps from transcription to the formation of functional miRNAs within the RNA-induced silencing complex [17] for mRNA targeting and gene expression regulation. *** The guide strand in the RISC complex is retained, while the passenger strand is removed and subsequently degraded. Abbreviations: DGCR8—DiGeorge Syndrome Critical Region 8; PACT—Protein Activator of the Interferon-Induced Protein Kinase; TRBP—TAR RNA-binding protein; DICER—Endoribonuclease Dicer; AGO2—Argonaute-2; RISC—RNA-induced silencing complex. This figure was sourced from BioRender.

Recent studies have significantly advanced our understanding of non-coding RNAs and their critical roles in numerous cellular functions [11]. MicroRNAs (miRNAs/miRs), which are endogenously transcribed RNA molecules of approximately 22 nucleotides, have emerged as key players in various biological processes. miRNAs are encoded by specific genes located in intergenic sequences or within intronic regions [12]. These regions are transcribed by RNA polymerase II or III to form a primary miRNA transcript (pri-miRNA) that is over 1000 nucleotides in length [13,14]. The pri-miRNA is then processed in the nucleus by a microprocessor complex, involving Drosha and DGCR8 proteins, to generate a precursor miRNA (pre-miRNA) of approximately 85 nucleotides [15,16]. This pre-miRNA is then exported to the cytoplasm via the exportin-5 and Ran-GTP protein complexes. In the cytoplasm, the Dicer enzyme and the TRBP/PACT protein complex further cleave and process the pre-miRNA to generate a mature 20–22 nucleotide miRNA duplex consisting of passenger and guide strands [16]. The guide strand, with lower thermodynamic stability at the 5′ end, is favored for integration into the Argonaute 2 (AGO 2) protein, resulting in the formation of an RNA-induced silencing complex [17] [15,16]. RISC targets are subjected to either translational repression or mRNA degradation, primarily through the formation of base pairs between the miRNA’s 5′ seed sequence and the 3′ untranslated region (UTR) of the target mRNA (Figure 1) [18,19].

The dysregulation of miRNAs is a common feature in various cancers, where they can act as either oncogenes (oncomiRs) or tumor suppressors, depending on their expression levels and the context of the cancer type [20]. MiRNAs influence several fundamental biological processes, including cell proliferation, differentiation, apoptosis, and angiogenesis, all of which are critical in cancer development and progression [20,21]. The dysregulation of miRNA expression can occur through various mechanisms, such as gene amplification, deletion, mutation, and epigenetic changes, which can lead to the aberrant expression of miRNAs and contribute to tumorigenesis [21,22]. Numerous studies have highlighted the significant impact that altered miRNA expression has on HCC pathogenesis, metastasis, and chemoresistance [23,24,25,26].

This review delves into the latest literature on the impact of miRNAs on oncogenic pathways in HCC and their implications for treatment outcomes. Furthermore, we offer insights into the current progress in the development of miRNA-based therapies for HCC treatment.

## 2. Role of miRNAs in Hepatocellular Carcinoma Cell Survival

Growth signaling autonomy, a hallmark of cancer, allows tumor cells to sustain proliferation independently of external growth factors. This autonomy is crucial for cancer progression, where it is associated with enhanced stemness, proliferation, and resistance to therapies [27,28]. In HCC, miRNAs are pivotal in regulating signaling pathways that contribute to cell survival and tumorigenesis. miRNA dysregulation characterized by an overexpression of oncomiRs and a reduction in tumor suppressor miRNAs leads to an excessive proliferation of growth signals and a diminished response to anti-growth and pro-apoptotic signals, ultimately permitting uncontrolled cell division [29].

### 2.1. miRNAs in Regulating Hepatocellular Carcinoma Cell Cycle and Proliferation

The tightly controlled regulation of the cell cycle involves essential components such as cyclin-dependent kinases (CDKs) like cyclin-D-dependent CDK4/CDK6 and cyclin-E-dependent CDK2. CDK inhibitors (CDKis) from the INK4 and Cip/Kip families, including p21Cip1, p27Kip1, and p57Kip2, also play crucial roles. Various signaling pathways such as RB-E2F, Hedgehog and Wnt, Ras-Raf-MEK-ERK, Hippo, and c-Myc are integral to this regulatory network [30]. When the cell cycle machinery malfunctions, leading to uncontrolled cellular proliferation, it becomes a significant feature of cancer [27]. miRNAs contribute to regulating these critical cell cycle pathways, affecting cellular processes [31].

In HCC, numerous tumor suppressor miRNAs inhibit cell cycle entry, including miR-9, miR-424-5p, miR-621, miR-125b-5p, the miR-29 family, and miR-450b-3p, which target HMGA2, E2F7, CAPRIN1, TXNRD1, RPS15A, and PGK1, respectively, leading to cell cycle arrest in the G0/G1 phase [32,33,34,35,36,37,38].

Overexpression of nucleolar and spindle associated protein 1 (NUSAP1), which is involved in mitosis, spindle assembly, and chromosome attachment [39], has been observed in HCC cells [40,41]. miR-193a-5p has been shown to induce G1 phase cell cycle arrest by targeting NUSAP1 in liver cancer cells, which in turn downregulates Cyclin E1, Cyclin D1, Cyclin B1, and Cyclin A2 and induces p21 expression [42].

Additionally, several miRNAs obstruct cell cycle progression in HCC by regulating the G1 to S phase transition (G1/S checkpoint). For example, miR-214 targets Wnt3a [43] and MELK [44] to decelerate HCC cell proliferation and induce G1 phase arrest. Ectopic overexpression of miR-0308-3p in HCC cells results in cell cycle blockade in the G1/S phase by downregulating CDK6 and Cyclin D1 [45]. Additionally, miR-217 reduced liver cancer cell proliferation and halted G1/S transition by targeting EZH2, Cyclin-D1 [46], MTDH [47], and KLF5 [48].

**Figure 2:**
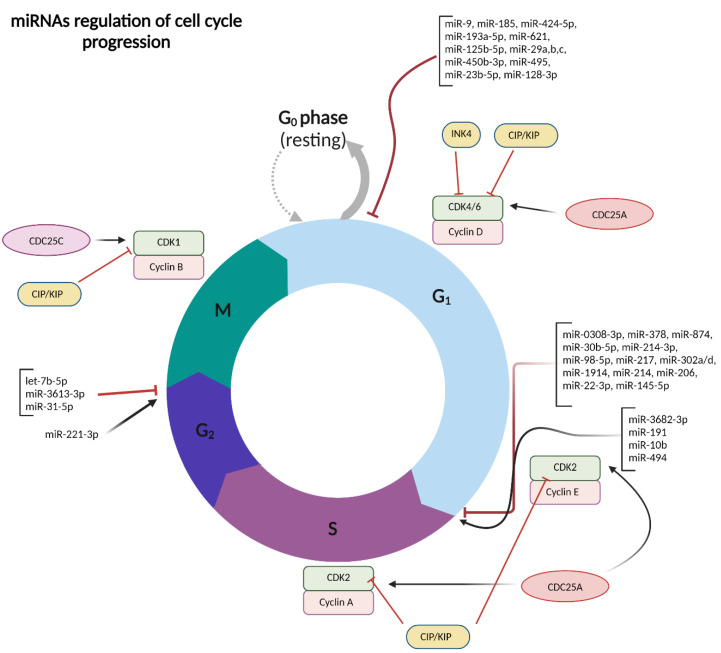
miRNAs regulate different stages of cell cycle progression in HCC, shedding light on the intricate roles of these molecules in modulating cell division and proliferation. Abbreviations: INK4—inhibitors of cyclin-dependent kinases; CIP/KIP—cyclin-dependent kinase (CDK) Inhibitory Proteins; CDC25C—Cell Division Cycle 25 C; CDC25A—Cell Division Cycle 25 A. This figure was sourced from BioRender.

Recent reports have highlighted the involvement of let-7-5p, miR-31-5p, and miR-3613-3p in regulating the later stages of the cell cycle, specifically by suppressing G2/M transition [49,50,51]. In contrast to cell cycle inhibitory miRNAs, pro-proliferative miRNAs, such as miR-494 [52], miR-191 [53], miR-3682-3p [54], miR-10b [55], and miR-221-3p [56] facilitate tumor growth and cell cycle progression in HCC (Figure 2).

### 2.2. Regulatory Role of miRNAs in Hepatocellular Carcinoma Cell Death Pathways

Multiple stress conditions can activate various death signaling pathways, including necrosis, apoptosis, necroptosis, mitoptosis, ferroptosis, pyroptosis, and autophagy [57]. Apoptosis, necroptosis (programmed necrosis), and autophagy have been extensively studied in the context of liver cancer, and dysregulation of these death signaling pathways play a crucial role in drug resistance and treatment failure in HCC.

#### 2.2.1. Apoptosis Related miRNAs

Apoptosis, a programmed cell death process, is facilitated by a family of proteases known as caspases (cysteinyl aspartate-specific proteases) [17]. This type of cell death can be initiated by intrinsic signals, such as genotoxic stress, or extrinsic signals, such as the binding of ligands to cell surface death receptors (DRs), including TNF-R1, CD95 (Fas), TRAIL-R1 (DR4), TRAIL-R2 (DR5), DR3, and DR6 [58].

miRNAs modulate apoptotic events by acting as pro- or anti-apoptotic miRNAs. For example, members of the BCL-2 family of genes, the chief mediators of apoptosis, are regulated by multiple miRNAs including miR-378 [59], miR-9-5p [60], miR-448 [61], and miR-133b [62].

##### 2.2.1.1. Pro-Apoptotic miRNAs in Hepatocellular Carcinoma

miRNA profiling of HCC tissues revealed downregulation of pro-apoptotic miRNAs in cancerous cells [63]. Corroborating these observations, studies have shown that treatment of HCC cells with miR-133b mimics, a pro-apoptotic miRNA, induces apoptotic cell death by enhancing the activities of caspase-3/-8 and increasing the Bax/Bcl-2 protein expression ratio by regulating the EGFR/PI3K/Akt/mTOR axis [62]. Similarly, miR-22-3p promotes apoptosis by inhibiting the Akt/PI3K pathway via direct suppression of AKT2 protein expression [64]. Furthermore, Wang et al. recently reported that miR-206 suppresses c-MET expression, thereby reducing the malignant behavior of HCC cells and stimulating apoptosis [65].

**Figure 3:**
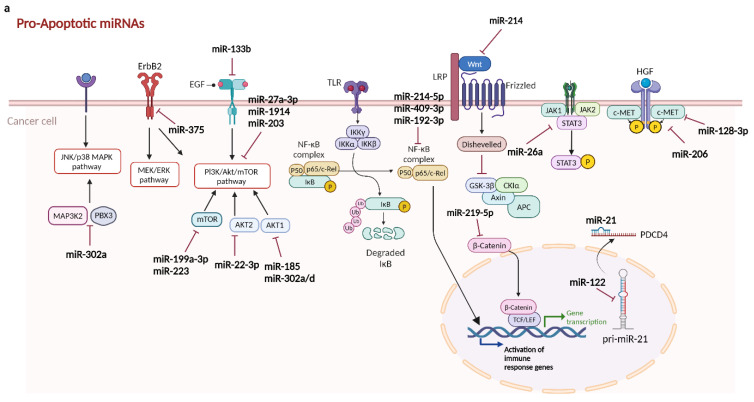

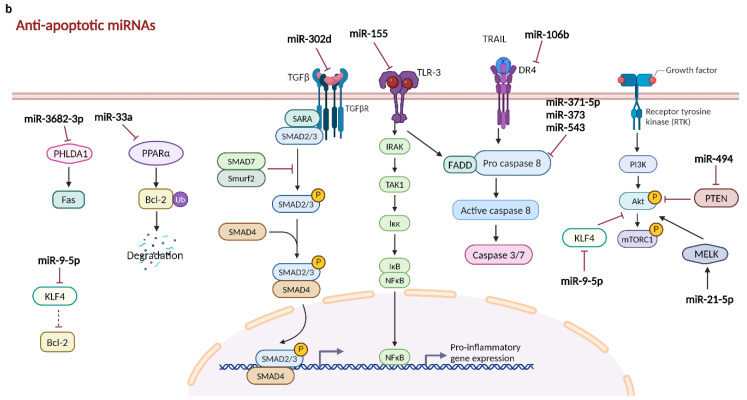
miRNAs play a pivotal role in maintaining the delicate balance between cell survival and death by modulating the activity of key pathways such as PI3K/AKT/mTOR, MAPK, TGF-β, Wnt/β-catenin, and JAK/STAT. Pro-apoptotic miRNAs (**a**): Some miRNAs act as pro-apoptotic regulators targeting anti-apoptotic genes in signaling pathways, promoting programmed cell death. Anti-apoptotic miRNAs (**b**): Conversely, certain miRNAs serve as anti-apoptotic regulators by targeting pro-apoptotic genes, inhibiting apoptosis and promoting cell survival. Abbreviations: CKIα—Casein Kinase I alpha; GSK-3β—Glycogen Synthase Kinase 3 beta; TCF—T Cell Factor; LEF—Lymphoid Enhancer-Binding Factor; PDCD4—Programmed Cell Death Protein 4; FADD—Fas-Associated protein with Death Domain; PHLDA1—Pleckstrin Homology-like Domain, Family A, Member 1; Smurf2—SMAD-specific E3 ubiquitin protein ligase 2; IRAK—Interleukin-1 Receptor-Associated Kinase; TAK1—Transforming growth factor Beta-Activated Kinase 1. This figure was sourced from BioRender.

Importantly, the JAK1/STAT3 signaling pathway has emerged as an important survival mechanism in HCC, promoting damage repair, cellular renewal, and rejuvenation within the cirrhotic microenvironment, thereby exerting an anti-apoptotic effect on liver cancer cells [66]. miR-26a has been found to be pro-apoptotic in HCC cells by directly targeting JAK1 to modulate its expression, leading to increased apoptosis [67]. Moreover, the JNK and MAPK pathways are closely associated with the stress response and apoptosis [68,69,70], and notably MAP3K2 is upregulated in many cancers [71,72,73]. Recent evidence suggests that miR-302a exerts antiproliferative and pro-apoptotic effects in HCC cells by targeting both MAP3K2 and PBX3 (Figure 3a) [74].

##### 2.2.1.2. Anti-Apoptotic miRNAs in Hepatocellular Carcinoma

Anti-apoptotic miRNAs are aberrantly overexpressed during tumorigenesis [63]. For example, miR-9-5p promotes HCC progression via inhibition of KLF4, thereby activating AKT/mTOR signaling, resulting in increased expression of the anti-apoptotic protein Bcl-2, and reduced expression of the pro-apoptotic protein Bax [60]. Furthermore, miR-33a facilitates HCC cell survival by inhibiting PPARα [75], a pro-apoptotic factor involved in the degradation of the Bcl-2 protein [76,77]. In HCC with underlying cirrhosis, miR-3682-3p was found to impair apoptosis and enhance cell survival by suppressing PHLDA1 expression and subsequent downregulation of Fas [54].

In viral hepatitis, activation of toll-like receptor 3 (TLR3) induces apoptosis of infected hepatocytes by promoting NF-kB transcription and activating caspase 8 [78,79]. miR-155 is often upregulated in HCC, contributing to the downregulation of TLR3, which is associated with apoptosis evasion and poor prognosis in these cancer cells [80]. The gold (Au) tagged antimiR-155 nanocomplexes (NCs) triggers TLR3-dependent apoptosis in HCC cells. These antimiR-155 NCs effectively silence miR-155 in HCC cells, inhibiting proliferation and migration while inducing apoptosis through TLR3 signaling [80].

miR-106b exhibits an anti-apoptotic role in HCC by negatively regulating DR4 [81]. The depletion of miR-106b increased sensitivity to TNF-related apoptosis-inducing ligand (TRAIL) treatment by upregulating DR4 expression and facilitating TRAIL-DR4 mediated cell death (Figure 3b) [81].

#### 2.2.2. Necroptosis Related miRNAs

Necroptosis (programmed necrosis) is a regulated form of lytic cell death that is triggered by various environmental stressors such as chemical and mechanical stress, osmotic shock, toxins, and viral and bacterial products [82,83]. A significant amount of experimental evidence has demonstrated that necroptosis contributes to tumor progression and metastasis by recruiting inflammatory cells, thereby promoting angiogenesis, proliferation, and invasiveness of cancer cells [84].

The role of miRNAs in regulating necroptosis in HCC has been underexplored in scientific literature. A study by Visalli et al. marks a significant step in this direction by identifying three specific miRNAs, namely miR-371-5p, miR-373, and miR-543, which exhibited abnormal overexpression in HCC tumor tissues and were found to drive necroptosis by inhibiting Casp-8 [85].

#### 2.2.3. Autophagy Related miRNAs

Autophagic cell death [86] is a regulated process in which cellular organelles and proteins are targeted for lysosomal degradation and categorized as type II cell death [87]. In tumorigenesis, ACD has dual context-dependent functional roles. ACD suppresses tumor initiation and malignant transformation [87] but also promotes the survival of tumor cells, metastatic progression, and drug resistance by providing substrates for cellular metabolism [88,89]. Autophagic flux involves the formation of autophagosomes, cargo degradation, and eventual recycling of breakdown products. The ULK1 complex, PI3K complex, ATG12-ATG5-ATG16L1, and LC3-PE, and lysosomal proteins such as LAMPs and cathepsins are involved in autophagosome maturation, elongation and closure, and cargo degradation, respectively [90,91]. Comprehending the functions of these proteins and their coordinated actions is crucial for understanding the molecular intricacies of autophagic flux in cancer cells.

##### 2.2.3.1. Pro-Autophagic miRNAs in Hepatocellular Carcinoma

As a key regulator of the autophagy pathway, AMP-activated protein kinase (AMPK) plays a role in various stages of autophagy, including initiation, autophagosome formation, and fusion with lysosomes [92]. Recent research suggests that miR-519d inhibits cell proliferation and promotes apoptosis and autophagy by targeting Rab10 expression and activating the AMP signaling pathway [93]. Experiments showed that restoring miR-519d in vivo suppressed tumor growth, with upregulation of autophagy-related genes e.g., Beclin1, Atg5, p53, and pro-apoptotic Bax, and downregulation of Rab10, mTOR, and Bcl-2 [93]. Additionally, miR-185 has been shown to have a tumor-suppressive effect in HCC cells by inducing autophagy through regulation of the Akt1 pathway [94].

##### 2.2.3.2. Anti-Autophagic miRNAs in Hepatocellular Carcinoma

In HCC cells, miR-181a-5p has been shown to attenuates autophagic flux by targeting Atg7 [95]. Additionally, Mig-6, a cytoplasmic protein that negatively regulates EGFR signaling [96], promotes apoptosis and inhibits the autophagic pathway through the upregulation of miR-193a-3p. Mechanistically, miR-193a-3p inhibits TGF-β2 protein expression, leading to decreased autophagy [97]. Another miRNA, miR-7, directly interferes with Atg5 and inhibits HCC metastasis [98]. Similarly, miR-30a reduces the expression of Beclin 1 and Atg5, resulting in autophagy inhibition and improved resistance to anoikis in HCC cells [99]. Furthermore, miR-26a/b has been identified as a direct inhibitor of ULK1 in HCC, impeding autophagic flux at an early stage and enhancing the sensitivity of cancer cells to chemotherapy [100].

Table 1 provides a comprehensive overview of miRNAs involved in the regulation of cell survival and proliferation in HCC.

## 3. miRNAs Regulatory Role in Tumor Cell Stemness

Cancer stem cells (CSCs) are a subset of cancerous cells within a tumor mass that have the ability to self-renew and generate new tumors, leading to relapse, metastasis, and resistance to radiation and chemotherapy [145]. Hepatic progenitor cells, hepatoblasts, and adult hepatocytes are potential sources of hepatic CSCs [146]. Specific antigenic markers (e.g., EpCAM, CD133, CD90, CD44, CD47, CD24, CD13, calcium channel α2δ1 isoform 5, K19, OV6, ABCG2, ALDH, and Hoechst dye efflux) can be used to identify and isolate liver CSCs [147,148,149]. These markers have been found to be expressed in different subsets of liver CSCs and have been used in combination to improve the sensitivity and specificity of CSC detection [150,151].

The stemness characteristics of cancer cells are maintained through various signaling pathways including Wnt/β-catenin, IL-6/STAT3, TGF-β, Notch, HH, Hippo, BMI1, NF-κB, PI3K/Akt/mTOR, and Ras/Raf/MAPK [152]. Other mechanisms contributing to the stemness of hepatic cancer cells include extracellular matrix (ECM) remodelling, epithelial-mesenchymal transition (EMT), hypoxia, epigenetic modifications, and autophagy [152]. miRNAs have been found to target important regulatory genes in these signaling pathways to acquire stem-like characteristics.

### 3.1. miRNAs Inhibiting Hepatocellular Carcinoma Stemness

Upregulation of the E2F family of transcription factors in CSCs plays a significant role in their self-renewal capacity, proliferation, aggressiveness, and resistance to chemotherapy and radiotherapy [153]. miRNA-302a/d inhibits the expression of E2F7 in liver CSCs, leading to attenuation of the AKT/β-catenin/CCND1 signaling pathway, repression of cell proliferation, and reduction in stemness characteristics [119].

Analysis of human HCC samples showed that CBX4 is abnormally upregulated in a subset of CSCs, marked by CD44+ CD133+ expression [154]. The miR-6838-5p/CBX4 axis affects liver CSC function via the ERK pathway with CBX4 as the target of miR-6838-5p [154]. In clinical HCC tissues, low miR-589-5p and high CD90 expression are correlated with vascular invasion and recurrence, and miR-589-5p negatively regulates CD90+ liver CSCs by suppressing MAP3K8 expression [155]. miR-148a and miR-148b suppress HCC progression and stemness, respectively, by targeting ACVR1 and NRP1 [156,157]. Additionally, Shi et al. reported that miR-296-5p mitigated EMT and stemness characteristics of HCC cells by modulating the Brg1/Sall4 axis and blocking NRG1/ERBB2/ERBB3/RAS/MAPK/Fra-2 signaling [158,159].

### 3.2. miRNAs Promoting Hepatocellular Carcinoma Stemness

Numerous miRNAs have been shown to play a role in promoting stem-like properties in HCC cells. Studies have demonstrated that the Oct4/miR-1246 axis can upregulate Wnt/β-catenin signaling in hepatic CD133+CSCs by targeting AXIN2 and GSK3β, leading to the accumulation of β-catenin [160]. Similarly, miR-5188 enhances the stemness phenotype by directly targeting FOXO1 and activating the Wnt/β-catenin cascade and EMT [161]. Another significant oncomiR in HCC is miR-106b-5p, which contributes to the stemness and aggressiveness of tumors by blocking PTEN expression and activating the PI3K/AKT pathway [162]. Table 2 lists the HCC stemness-regulating miRNAs and their underlying mechanisms.

## 4. miRNAs Regulatory Role in Hepatocellular Carcinoma Metastasis

**Figure 4:**
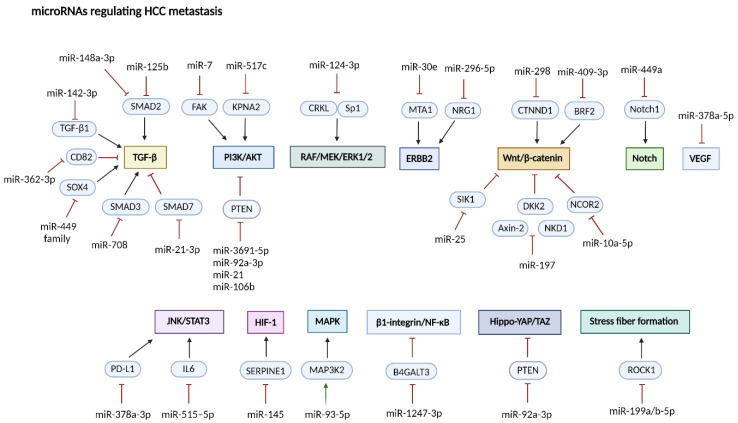
The schematic representation highlights how miRNAs involved in the metastatic process through modulation of key molecular pathways such as TGFβ, PI3K/AKT, Wnt/β-catenin, JNK/STAT, and Hippo/YAP, which are vital for cancer cell invasion and migration. Abbreviations: SERPINE1—Serpin Family E Member 1; B4GALT3—Beta-1,4 Galactosyltransferase 3; NCOR2—Nuclear Receptor Corepressor 2; NKD1—Naked Cuticle Homolog 1; DKK2—Dickkopf WNT Signaling Pathway Inhibitor 2; SIK1—Salt Inducible Kinase 1; CRKL—Crk-Like protein; NRG1—Neuregulin 1; MTA1—Metastasis-Associated Protein 1; BRF2—RNA Polymerase III Transcription Initiation Factor Subunit. This figure was sourced from BioRender.

The majority of cancer-related deaths (~90%) are attributable to metastasis rather than primary tumor growth [169]. The metastatic process involves several steps, including local tissue invasion, intravasation, survival in circulation, extravasation, and secondary site colonization [169,170]. During local invasion and metastatic dissemination, cancer cells undergo EMT, a process by which epithelial cells acquire a mesenchymal phenotype [27]. Dysregulation of miRNAs in HCC can affect key signaling pathways including TGF-β/Smad, MAPK, NF-kB, JAK/STAT, Hedgehog, Wnt/β-catenin, and Hippo-YAP transcriptional co-activators with TAZ, which are involved in EMT induction, and cancer progression (Figure 4) [171,172].

In HCC, invasion and metastasis occur at both intrahepatic and extrahepatic sites, indicating a highly aggressive tumor phenotype [173,174].

### 4.1. Anti-Metastatic miRNAs in Hepatocellular Carcinoma

Several miRNAs have been shown to have anti-metastatic effects in HCC, including miR-211-5p, miR-130a-3p, miR-193a-5p, miR-30a, miR-124, and miR-15a-3p [42,99,175,176,177,178,179].

Long-chain acyl-CoA synthetase 4 (ACSL4) is upregulated in HCC [180,181] and contributes to disease progression and poor prognosis by stabilizing c-Myc through the ERK/FBW7 axis [182]. miR-211-5p attenuates aggressive HCC features by directly regulating ACSL4 expression [175].

Recent findings have indicated that miR-130a-3p expression is reduced in HCC tumor tissues, and its restoration can inhibit cell proliferation, migration, and invasion by targeting the androgen receptor (overexpressed in approximately 37% of HCC cases [183]) and the subsequent decrease in β-catenin and Slug expression [176].

Research has linked irregular gene variants resulting from incorrect splicing to the promotion of cancer growth [184,185]. SF3B4, a subunit of the spliceosome complex, is highly dysregulated in HCC and serves as an early diagnostic biomarker [186]. miR-133b suppresses SF3B4, affecting downstream molecules, such as KLF4, KIP1, and SNAI2, potentially inhibiting tumor metastasis [126].

Focal adhesion kinase (FAK) plays a critical role in cell migration, metastasis [187], and angiogenesis [188,189], as a downstream mediator of angiogenic growth factor receptors. miR-7 inhibits the metastasis and invasion of HCC cells by modulating Snail-1, Slug, EGFR, TYRO3, and MMP-9. Additionally, it downregulates FAK expression, leading to downstream suppression of the Akt pathway [190]. Eukaryotic translation initiation factor 4A3 (eIF4A3) regulates mRNA splicing [191] and promotes tumor growth in HCC and other carcinomas [192,193,194]. miR-2113 inhibits cell migration and EMT by reducing the interaction between eIF4A3 and WDR66, a positive regulator of EMT, through downregulating WDR66 expression [195].

### 4.2. Pro-Metastatic miRNAs (metastamiRs) in Hepatocellular Carcinoma

Literature has consistently emphasized the essential role of miRNAs in various stages of tumor progression, such as transforming the tumor microenvironment and extracellular matrix, stimulating neoangiogenesis, and facilitating tumor cell invasion, metastasis, and colonization. For example, miR-18a [196,197], miR-25 [198], miR-106b-5p [199], miR-183-5p [138], and miR-376c-3p [200] are noteworthy miRNAs that act as pro-metastatic miRNAs in HCC. These miRNAs target genes involved in cancer cell migration and invasion, including Bcl2L10, KLF4, Fbxw7, FOG2, PDCD4, and ARID2.

Moreover, miR-93-5p promoted liver cancer cell metastasis by activating the MAP3K2/p38-JNK/p21 signaling pathway [201]. ChIP analysis of HepG2 HCC cells revealed a positive feedback loop between miR-93-5p, MAP3K2, and c-Jun, where c-Jun targets the miR-93-5p promoter to enhance its transcription [201]. Table 3 classifies metastasis-related miRNAs in HCC.

## 5. Exosomal miRNAs in Hepatocellular Carcinoma Progression

The complex process of cancer cell metastasis requires malignant cells to evade death signals and nullify immune responses to facilitate local invasion and colonization at secondary sites [264]. In solid cancers, tumor microenvironment (TME)-embedded cancer cells represent a complex and dynamic stroma that consists of fibroblasts, endothelial cells, mesenchymal stem cells, adipocytes, and immune cells (e.g., macrophages, neutrophils, dendritic cells, and lymphocytes). Moreover, blood and lymph vessels as well as non-cellular components, including cytokines, extracellular vesicles (EVs), and extracellular matrix (ECM), are among the other TME components [265]. It is well established that an intercellular communication network exists between a primary tumor and its stroma, driven by various growth factors, cytokines, chemokines, and EVs to sustain cancer cell survival and promote invasion and metastasis [266].

Exosomes are EVs which develop from the endosomal plasma membrane, are 30–150 nm in size and serve as cargo carriers that transport molecules, including DNA, mRNA, miRNAs, proteins, and lipids [267]. Accordingly, exosome-delivered miRNAs can trigger specific signaling pathways in recipient cells to modulate processes involved in tumor development and invasion, such as EMT, angiogenesis, stemness, chemoresistance, and immune responses [268].

### 5.1. EMT Related Exosomal miRNAs in Hepatocellular Carcinoma

Exosomal miRNAs have emerged as key players in the progression of HCC [269]. For instance, highly metastatic HCC cells secrete miR-92a-3p in exosomes and promote EMT and metastasis of neighbouring less metastatic cancer cells by suppressing PTEN and activating the Akt/Snail signaling pathway [244].

Hypoxia is a common condition in solid tumors, including HCC, where there is upregulation of specific hypoxia-inducible factors (HIFs), leading to cancer progression and therapeutic resistance [270,271]. Tian et al. reported that HIF binding to the promoter regions of exosomal miRNAs miR-21 and miR-10b induces a metastatic phenotype in HCC by upregulating vimentin and reducing the expression of PTEN and E-cadherin [272]. Another study found that, under hypoxic conditions, HCC cells secrete exosomes rich in miR-1273f, which augments proliferation, migration, invasion, and EMT phenotypes in recipient HCC cells by direct inhibition of LHX6 [260].

However, certain exosomal miRNAs act as tumor suppressors, inhibiting cancer development and progression. For example, exosomal miR-125b has been shown to exert anti-metastatic effects by inhibiting MMPs, targeting SMAD2, and disrupting the TGF-β1/SMAD signaling pathway, which collectively leads to reduced EMT [209]. Notably, exosomal miR-125b is a potential early biomarker for detecting extrahepatic metastasis in HCC, given that lower serum exosomal miR-125b levels are associated with an increased risk of metastasis in cancer patients [209].

### 5.2. Exosomal miRNAs Involved in Non-EMT Related Hepatocellular Carcinoma Progression and Metastsis

Emerging data suggest that metastasis is a multi-directional process where circulating tumor cells (CTCs) can settle in both distant organs and the primary tumor itself, a phenomenon known as “tumor self-seeding” [273]. Tumor-derived inflammatory cytokines, such as interleukin (IL)-6 and IL-8 attract CTCs [274,275]. Exosomal miR-25-5p secreted from primary HCC cells stimulates the trans-endothelial mobility and tumor self-seeding potency of CTCs by downregulating the LRRC7 gene [258]. Another study found that exosome-derived miR-25-5p released from a highly metastatic HCC cell line (CSQT-2) increases the aggressiveness of cancer cells by inhibiting SIK1 [257], thereby activating the Wnt/β-catenin signaling pathway. Moreover, loss of liver glycine N-methyltransferase (GNMT) induces hepatic steatosis and disease progression in HCC [276]. Studies have shown that exosomal miR-224 drives tumor progression and invasiveness by directly targeting GNMT, and the serum expression of exosomal miR-224 is higher in HCC patients than in healthy individuals [261].

## 6. miRNAs in Tumor Microenvironment Remodelling

**Figure 5:**
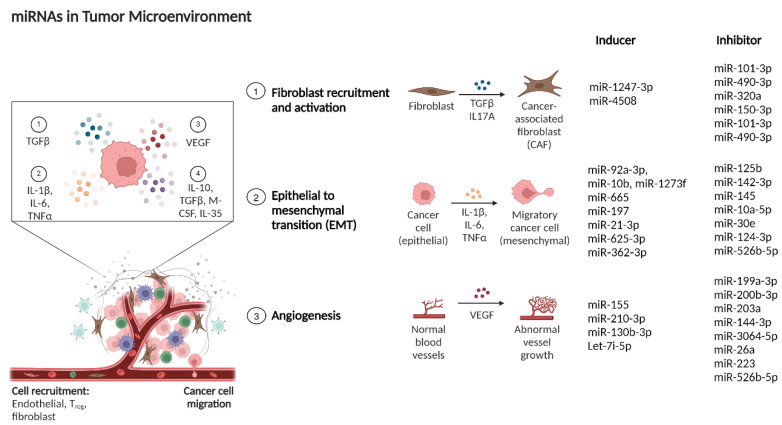

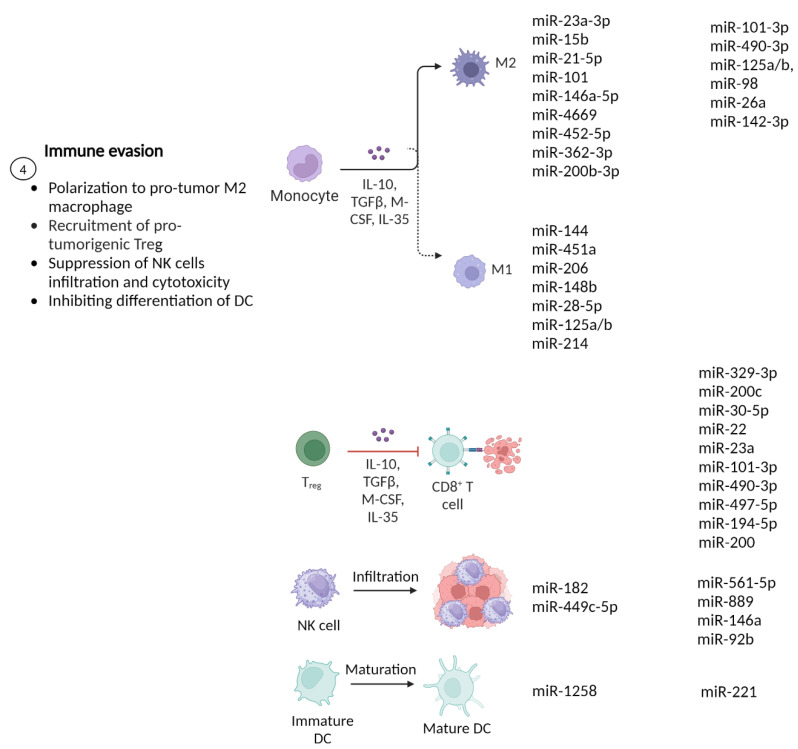
miRNAs influence complex interactions between cancer cells and their environment, modulating processes like fibroblast activation, EMT, angiogenesis, and immune evasion. Abbreviations: TGFβ—Transforming Growth Factor beta; VEGF—Vascular Endothelial Growth Factor; CSF—Colony Stimulating Factor; NK—Natural Killer; DC—Dendritic Cell. This figure was sourced from BioRender.

Several studies have demonstrated the importance of miRNAs in shaping the tumor microenvironment (TME) and promoting cancer progression (Figure 5) [277]. The following sections summarize the latest research on the role of miRNAs in the TME and provide insights into HCC management.

### 6.1. miRNAs Regulating Cancer-Associated Fibroblasts

Cancer-associated fibroblasts (CAFs) are activated fibroblasts with high expression of alpha-smooth muscle actin (αSMA) and fibroblast activation protein (FAP) and are located near neoplastic lesions [278,279]. CAFs secrete factors such as TGF-β, hepatocyte growth factor (HGF), stromal cell-derived factor 1 (SDF-1), and IL-1β to mediate immune suppression, extracellular matrix remodelling, maintenance of tumor stemness, angiogenesis, and chemoresistance [280].

In a hypoxic environment, tumor cells release miR-4508 within exosomes, which in turn activates pulmonary fibroblasts to yield a permissive pre-metastatic niche in the lungs of mice by targeting RFX1 [281]. Furthermore, knockdown of RFX1 induces an activated fibroblast phenotype in the pre-metastatic niche via its effect on the p38 MAPK-NF-kB signaling pathway [281]. In addition, miR-4508 is also reported to be elevated in plasma exosomes of patients with HCC after trans-arterial chemoembolization (TACE). Similarly, Fang et al. found a correlation between high serum levels of exosomal miR-1247-3p and lung metastasis in HCC patients [263]. Specifically, researchers found that in the metastatic lung niche, HCC cells secrete exosomal miR-1247-3p, which leads to the conversion of normal fibroblasts into CAFs through direct inhibition of B4GALT3 and activation of β1-integrin/NF-κB signaling in fibroblasts [263].

Zhang et al. found that miR-320a expression was reduced in CAF-derived exosomes compared to normal fibroblasts, and ectopic miR-320a expression impaired the migration, invasion, and EMT phenotype of cancer cells via suppression of the MAPK/ERK1/2-CDK2 axis and direct inhibition of PBX3 [177]. Moreover, reduced miR-150-3p levels have also been reported in CAF-derived exosomes, which upon transfer to HCC cells, inhibit their proliferation and metastatic capabilities [282]. Clinically, poor miR-150-3p expression in HCC tissues is a significant risk factor for recurrence [282].

Another study reported that HCC cell-derived exosomal miR-21 directly targets PTEN, leading to the activation of hepatic stellate cells (HSCs) into CAFs by triggering PDK1/AKT signaling and lipogenesis [259]. Consistently, high serum levels of exosomal miRNA-21 were associated with higher CAF activation and tumor vasculature density in patients with HCC [259]. The expression of miR-101-3p and miR-490-3p have been reported to be downregulated in HCC CAFs [283]. TGFBR1 is a common target gene of these miRNAs, and its expression is positively correlated with the infiltration of myeloid-derived suppressor cells, regulatory T cells, and M2 macrophages, which create an immunosuppressive TME and facilitate HCC progression [283].

### 6.2. miRNAs in Regulating Tumor-Associated Macrophages

Tumor-associated macrophages (TAMs) are an M2-polarized subtype of macrophages that are considered key factors in the development of an immunosuppressive TME that supports HCC progression and metastasis [284]. Therapeutic strategies targeting TAMs in animal models of HCC have efficiently attenuated tumor growth [284,285]. Several studies have highlighted the important roles of specific exosomal miRNAs in modulating TAM infiltration and influencing HCC aggressiveness and metastasis (Figure 5).

Nakano et al. demonstrated that in patients with post-transplant HCC recurrence, high exosomal miR-4669 levels contribute to an immunosuppressive TME by inducing M2 macrophage polarization, which enhances tumor aggressiveness [286]. Similarly, exosomal miR-452-5p secreted by HCC cells was found to promote M2 macrophage polarization by targeting TIMP3, thereby contributing to HCC progression and metastasis [287].

In contrast, the miR-144/miR-451a cluster promotes M1 macrophage polarization and antitumor activity in HCC by targeting HGF and MIF [288].

Furthermore, Chen et al. found that deficiency of miR-125a and miR-125b in TAM-derived exosomes promoted the proliferation, stem cell properties, and metastatic capacity of HCC cells, and re-expression of miR-125a/b in HCC cells suppressed the growth and sphere formation ability of liver cancer cells by targeting CD90 [163].

Importantly, Ke et al. showed that a lack of miR-148b leads to the overexpression of CSF1, which induces TAM infiltration and promotes HCC metastasis through CSF1/CSF1R signaling [289]. Moreover, Zhou et al. demonstrated that a reduction in miR-28-5p levels increases HCC growth and metastasis via IL-34-mediated TAM infiltration [290].

### 6.3. miRNAs in Regulating Natural Killer Cells, T Cells, and Dendritic Cells

Immune checkpoints (e.g., programmed death protein 1 (PD-1)) play a crucial role in regulating immune responses, and miRNAs can be used to target and modulate the expression of immune checkpoints on the surface of natural killer (NK) cells and T cells or that of their ligands on cancer cells, leading to potential applications in antitumor immunotherapy [291]. PD-1 is a cell surface receptor found on various immune cells that promotes self-tolerance and downregulates the immune response. The PD-1 ligand, PD-L1, is highly expressed in cancer cells and can inhibit the proliferation of immune cells, leading to immune tolerance within the tumor microenvironment [292].

In HCC models, miR-223 was reported to exert an indirect downregulatory effect on PD-1 and PD-L1 through the suppression of the HIF-1α-driven CD39/CD73-adenosine pathway, thereby impeding HCC progression [293]. Wang et al. reported that KDM1A interacts with MEF2D to increase PD-L1 expression in HCC cells, and miR-329-3p targeting of KDM1A inhibits tumor-induced immunosuppression and sensitizes HCC cells to T cell-mediated cytotoxicity, by modulating PD-L1 expression [294].

Sun et al. found that miR-200c and PD-L1 expression were inversely correlated in HBV-induced HCC, as miR-200c directly targets the 3ʹ-UTR of PD-L1 which leads to a reversal of CD8+ T cell exhaustion [295]. However, HBV-induced STAT3 activation triggers SALL4 expression, which in turn suppresses miR-200c transcription, suggesting that the HBV-pSTAT3-SALL4-miR-200c axis regulates PD-L1 expression [295].

Wan et al. recently highlighted the potential of miR-22 as a therapeutic target for modulating immune responses in HCC [296]. miR-22 delivery via adeno-associated virus serotype 8 significantly affected the function of hepatocytes and T cells by silencing HIF-1α and enhancing retinoic acid signaling in both cell types. miR-22 was observed to decrease the abundance of IL17-producing T cells and inhibit IL17 signaling in the liver, thereby promoting the expansion of cytotoxic T cells and reducing the population of regulatory T cells [296].

CXCL10 plays a crucial role in CD8 + T and NK cell trafficking [297]. According to experimental evidence, DPP4 is involved in the N-terminal truncation of CXCL10, which limits T-cell and NK cell migration [298]. Huang et al. reported that the miR-30-5p/Snail/DPP4/CXCL10 axis influences the HCC-immune microenvironment by enhancing the stability of CXCL10 and improving CD8+ T-cell infiltration [299].

Chen et al. showed that CX3CL1 stimulates the chemotactic migration of CX3CR1+ NK cells through STAT3 signaling, and miR-561-5p promotes tumor growth and lung metastasis by suppressing CX3CL1 expression, leading to low NK cell infiltration and poor prognosis in patients with HCC [4].

In a HCC mouse model, miR-1258 inhibited tumor development and metastasis by stimulating TLR7/8 expression and inducing the antitumor activity of dendritic cells (DCs) and NK cells [300].

Xie et al. demonstrated that the expression of MICB in tumor cells was negatively correlated with miR-889 in HCC tissues and that miR-889 upregulation reduced HCC cell susceptibility to NK lysis [301].

Table 4 presents a comprehensive list of various miRNAs involved in the modulation of the tumor microenvironment in HCC.

### 6.4. miRNAs Involved in Tumor Angiogenesis Regulation

Tumor neovascularization, also referred to as tumor angiogenesis, is a sophisticated biological process that develops new blood vessels within the tumor microenvironment [314], and serves as an essential step for cancer progression and metastasis. As a pathological condition, an imbalance between angiogenic stimulators and inhibitors results in robust neovascularization in tumors [315]. Several studies have explored angiogenesis-related miRNAs in various human carcinomas (Figure 5).

#### 6.4.1. Anti-Angiogenic miRNAs in Hepatocellular Carcinoma

The interaction between HCC cells and VECs is fundamental for the construction of tumor blood vessels. Research has identified that miR-199a-3p disrupts this interaction by directly inhibiting VEGFA expression in HCC cells and decreasing VEGFR1 and VEGFR2 expression in VECs [316]. HOXA3, HOXB3, and HOXD3 control angiogenesis, and HOXD3 directly targets the VEGFR promoter region [317]. miR-203a suppresses HCC cell invasion, metastasis, and angiogenesis by inhibiting the VEGFR pathway and targeting HOXD3 expression [318]. The PI3K/AKT pathway and its downstream mediator SGK3 facilitate tumor angiogenesis [319]. Wu et al. showed that miR-144-3p is a critical regulator of this pathway by reducing SGK3 expression and downregulating VEGF2, thereby inhibiting the angiogenic capabilities of HCC cells [320].

The ERG plays an essential role in endothelial differentiation and angiogenesis [321]. Hepatocytes release exosomal miR-200b-3p, which attenuates angiogenesis by blocking endothelial ERG expression [322]. Furthermore, downregulation of miR-3064-5p correlates with high angiogenesis potential in HCC tissues by inhibiting VEGFA and angiogenin while inducing endostatin and MMP12 expression through the FOXA1/CD24/Src pathway [323]. This relationship underscores the intricate balance of pro- and anti-angiogenic factors governed by microRNAs in the tumor microenvironment.

#### 6.4.2. Pro-Angiogenic miRNAs in Hepatocellular Carcinoma

Researchers have discovered several pro-angiogenic miRNAs that play roles in various cancers by specifically regulating the VEGF/HIF-1 pathway [324]. One of the key factors in this process is tumor hypoxia, which activates the hypoxia-inducible factors HIF-1 and HIF-2, which in turn trigger the transcription of genes involved in angiogenesis [325]. For instance, in HCC cells under hypoxic conditions, both cellular and exosomal miR-155 were found to be overexpressed and to have the ability to induce tube formation in human umbilical vein endothelial cells [326]. Clinical investigations have also demonstrated a positive correlation between miR-155, HIF-1α, and VEGF expression in HCC tissue [326].

Conversely, there are genes and factors with anti-angiogenic properties that promote vascular stability. HOXA5 inhibits angiogenesis by increasing the expression of anti-angiogenic factors (e.g., p53) and decreasing the expression of pro-angiogenic factors (e.g., HIF-1α and VEGFR2), thereby promoting vascular stability [327]. Studies have shown that miR-130b-3p is a pro-angiogenic miRNA as it can suppress HOXA5 expression, leading to enhanced angiogenic capacity in HCC cells [328].

Smad4 is another important factor in the regulation of angiogenesis, exerting its anti-angiogenic properties via the suppression of VEGF expression and upregulation of the angiogenesis inhibitor TSP-1 [329]. Lin et al. reported that exosomal miR-210-3p facilitates intercellular communication between HCC and endothelial cells by inhibiting Smad4 and STAT6 in vascular endothelial cells, thereby promoting angiogenesis [330].

Table 5 provides an overview of angiogenesis-related miRNAs in HCC.

## 7. miRNAs in Hepatocellular Carcinoma Drug Resistance

**Figure 6:**
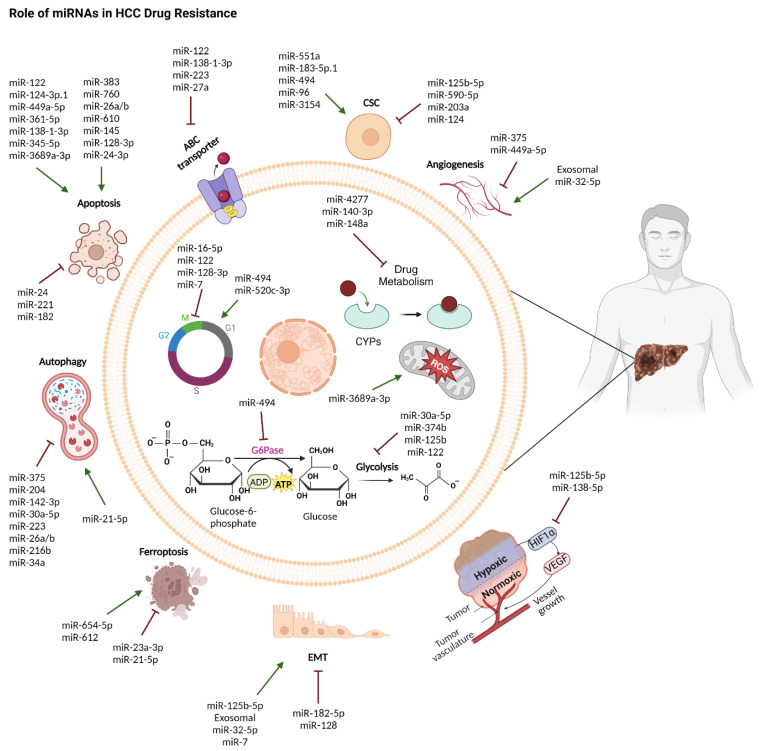
miRNAs play multifaceted roles in orchestrating chemoresistance in HCC, influencing various cellular processes like proliferation, EMT, autophagy, apoptosis, ferroptosis, metabolism, stemness, ABC transporter activity, hypoxia, and ROS regulation. Abbreviations: CSC—Cancer Stem Cells; ABC—ATP-Binding Cassette transporter; CYPs—Cytochrome P450 enzymes; G6Pase—Glucose-6-phosphatase. This figure was sourced from BioRender.

Tumor heterogeneity and poor survival rates in HCC pose significant clinical challenges. As chronic liver disease progresses to HCC, changes in TME can have a major impact on drug metabolism and response to therapy [335]. Drug resistance remains a major obstacle in HCC management, stemming from multiple factors, including enhanced drug efflux, reduced drug uptake, intracellular detention, high drug metabolism, aberrant apoptotic and autophagic signaling, TME remodelling, and the acquisition of stem cell-like features [336]. Recently, researchers have focused on miRNAs and their roles in the development of chemoresistance in HCC (Figure 6) [337]. This section reviews the current understanding of the role of miRNAs in drug sensitivity in HCC and examines the potential mechanisms and clinical implications.

### 7.1. miRNAs in Chemotherapy Response

The most common chemotherapy intervention for unresectable HCC is transcatheter arterial chemoembolization (TACE) using doxorubicin, cisplatin, and 5-fluorouracil (5-FU). miRNAs can modulate drug responses through various mechanisms, including the regulation of autophagy, membrane transporters, EMT, CSCs, TME, and metabolic reprogramming (Figure 6) [337].

#### 7.1.1. miRNAs Promoting Hepatocellular Carcinoma Cells Sensitivity to Chemotherapy

In a study involving four distinct HCC cohorts, researchers discovered that the miR-125b/HIF-1α axis plays a critical role in determining HCC cell sensitivity to TACE therapy [338]. This study revealed that miR-125b directly reduces the translation of HIF-1α and disrupts the autocrine HIF-1α/PDGFβ/pAkt/HIF-1α loop by targeting the PDGFβ receptor. Moreover, the loss of miR-125b and increased HIF-1α expression upregulate CD24 and erythropoietin, resulting in the enrichment of doxorubicin-resistant CD24+ cancer stem cell population [338].

Other studies have demonstrated that miR-26a/b and miR-223 suppress doxorubicin-induced autophagic flux by targeting ULK1 and FOXO3a, respectively, resulting in an improved sensitivity to doxorubicin and increased apoptosis [100,339].

Additionally, the HIF-2α-MALAT1-miR-216b axis regulates multidrug resistance in HCC cells by modulating the expression of the autophagosome marker LC3-II and autophagy [340]. MALAT1 is an oncogenic long non-coding RNA whose expression is induced by HIF-2α, which then downregulates miR-216b in HCC cells. MALAT1 siRNA and miR-216b mimics inhibit LC3-II levels and autophagy, while enhancing 5-FU-induced apoptosis [340].

Moreover, miR-361-5p and miR-610 have been shown to enhance cisplatin sensitivity by reducing the expression of MAP3K9 and HDGF in HCC cells [341,342]. The RNA-binding protein MSI1 is upregulated in malignancies and modulates cancer cell proliferation by influencing the Notch, Wnt, and Akt signaling pathways [343]. Interestingly, miR-10a-5p has been found to promote cisplatin sensitivity by downregulating MSI1 and impairing the Akt signaling pathway in HCC [344].

#### 7.1.2. miRNAs Promoting Hepatocellular Carcinoma Cells Resistance to Chemotherapy

Myocyte-specific factor 2C (MEF2C) plays an essential role in regulating cell differentiation, stemness, proliferation, and migration [345,346]. In a study by Kang et al., cells expressing miR-551a displayed resistance to 5-FU induced cell death and exhibited enhanced survival and sphere formation after 5-FU treatment, with these effects attributable to the miR-551a targeting of MEF2C [347].

Furthermore, miR-182 levels were significantly increased in HCC patients undergoing cisplatin-based chemotherapy, and the upregulation of miR-182 enhanced cell viability during cisplatin treatment by targeting TP53INP1 [348].

Table 6 outlines the various miRNAs that play a role in the response of HCC to chemotherapy.

### 7.2. miRNAs in Targeted Therapy Response

In recent years, there has been a growing interest among researchers in studying the influence of miRNA dysregulation on the efficacy of targeted therapies, such as sorafenib and lenvatinib, in the context of HCC. Sorafenib, a first-line targeted therapy approved for advanced HCC treatment (since 2008) [365], functions by suppressing EGFR 1–3 and PDGFR-II, acting as an anti-angiogenic agent [366]. However, sorafenib only provides a modest improvement in patient survival of approximately three months, primarily owing to the development of resistance [367]. Another targeted therapeutic agent, lenvatinib, was approved by the FDA in 2018 [368]. It is an oral multikinase inhibitor that effectively blocks various receptors, including VEGFRs, FGFRs, RET, C-kit, PDGFR-α, and PDGFR-β, and inhibits downstream signaling pathways involved in tumor angiogenesis and cancer cell proliferation [369].

The mechanisms underlying sorafenib and lenvatinib resistance in HCC are not yet fully understood. An in-depth understanding of miRNAs and their impact on the development of targeted therapy resistance is important for the development of innovative therapeutic strategies for HCC. Targeting these miRNAs or their downstream signaling pathways could potentially restore the sensitivity of targeted therapy and enhance treatment efficacy [370].

#### 7.2.1. miRNAs Improving Sorafenib Response

Sorafenib resistance in hepatocellular carcinoma (HCC) involves a complex interplay of molecular mechanisms. Kabir et al. [371] identified a miR-7/TYRO3 axis that regulates the growth and invasiveness of sorafenib-resistant HCC cells, suggesting a potential therapeutic role for miR-7. Several other miRNAs have been identified as potential enhancers of the efficacy of sorafenib treatment in HCC cells. For example, miR-449a-5p improves sorafenib-induced apoptosis and reduces angiogenesis in HCC cells by downregulating PEA15, PPP1CA, and TUFT1, and modulating the Akt/ERK signaling pathway [372]. Another miRNA, miR-4277, sensitizes HCC cells to sorafenib treatment by targeting CYP3A4 and reducing its metabolism and clearance [373].

Autophagy, a major mechanism underlying acquired sorafenib resistance, can be induced by sorafenib treatment via regulation of various targets [374]. Importantly, restoration of miR-30a-5p by hydroxychloroquine treatment has been found to improve sorafenib response by suppressing autophagy through ATG5 and Beclin-1 [375]. Furthermore, the miR-30a-5p/CLCF1 axis has been implicated in regulating sorafenib resistance by directly targeting CLCF1, modulating PI3K/Akt signaling, and attenuating aerobic glycolysis in sorafenib-resistant HCC cells [376].

miR-204 has also been identified as a regulator of autophagy and the sorafenib response by suppressing ATG3 in HCC [377]. Moreover, ectopic miR-142-3p overexpression sensitizes HCC cells to sorafenib by decreasing sorafenib-induced autophagy and enhancing sorafenib-induced apoptosis by targeting ATG5 and ATG16L1 [378].

miR-375 also plays a pivotal role in reducing the resistance of HCC cells to sorafenib by inhibiting autophagy and exerting anti-angiogenic effects through targeted regulation of SIRT5 and PDGFC [332,379]. Additionally, sorafenib administration has been shown to induce miR-375 expression in hepatoma cells via the transcription factor ASH1. The expression of miR-375 is decreased in sorafenib-resistant cells, but restoring its levels can partially re-sensitize cells to sorafenib, primarily through the degradation of the AEG-1 gene [332].

In a study by Lin et al., liver X receptor-α activation with its agonist (GW3965) was found to induce the transcription of miR-378a-3p, which improves sorafenib response by regulating IGF1R expression and inhibiting ERK/PI3K signaling [380].

The pregnane X receptor (PXR), a member of the nuclear receptor superfamily, regulates genes involved in xenobiotic metabolism and drug resistance, such as CYP450 and ATP-binding cassette transporters [381], and studies have shown that sorafenib therapy triggers PXR expression in HCC cells, resulting in increased drug clearance and reduced sensitivity [382]. However, miR-140-3p and miR-148a can target PXR expression, enhance sorafenib retention in HCC cells, and restore sensitivity [383,384].

Another study identified FOXO3a, a crucial transcription factor in the cellular stress response, as a significant player in miR-124-3p.1 mediated sorafenib efficacy in HCC cells [385]. miR-124-3p.1 enhances sorafenib-induced apoptosis by increasing the nuclear localization of FOXO3a and maintaining its dephosphorylation and acetylation through the targeting of upstream regulators Akt2 and SIRT1 [385]. Additionally, Pei et al. found that PAK5 phosphorylates β-catenin, facilitating its translocation to the nucleus and promoting ABCB1 transcription. Importantly, miR-138-1-3p inhibits PAK5, reduces β-catenin/ABCB1 signaling, and improves sorafenib response [386]. ADAM-17, which is involved in the processing and activation of Notch proteins [387], is targeted by miR-3163, which enhances sorafenib sensitivity by suppressing Notch signaling activation [386]. Moreover, miR-345-5p targets TOP2A mRNA and improves the effects of sorafenib treatment in HCC by inducing apoptosis [388]. In a separate discovery, miR-3689a-3p emerged in CRISPR/Cas9 screening as a key miRNA that increased sorafenib sensitivity in HCC cells. miR-3689a-3p targets CCS, disrupting the copper balance and SOD1 function, ultimately leading to sorafenib-induced HCC cell death [389].

#### 7.2.2. miRNAs Inducing Sorafenib Resistance

Lu et al. discovered that miR-23a-3p is highly expressed in sorafenib-non-responder HCC patients [390], and further proteomic analysis revealed that miR-23a-3p blocks ferroptosis by suppressing the expression of ACSL4, an essential enzyme for ferroptosis [391]. In another study, miR-125b-5p induced sorafenib resistance by promoting Snail-mediated EMT via ATXN1 inhibition [392].

Pollutri et al. found that miR-494 expression was significantly upregulated in HCCs with stem cell-like characteristics in both humans and rats, and high miR-494 levels were linked to a poor sorafenib response [52]. miR-494’s oncogenic function is attributed to its targeting of p27, PUMA, and PTEN genes, as well as its activation of the mTOR pathway in HCC cell lines [52]. Furthermore, miR-494 contributes to metabolic plasticity and survival of HCC cells by promoting glycolysis by targeting G6pc and activating the HIF-1α pathway [393]. Elevated serum levels of miR-494 have been linked to sorafenib resistance in preclinical models and patients with HCC. Furthermore, the use of combination therapy with antagomiR-494 and sorafenib or 2-deoxy-glucose has demonstrated improved anticancer effects in HCC cells [393].

A recent study found that high expression of miR-21-5p and USP24 is associated with cancer progression and drug resistance in HCC by promoting autophagy via USP24-mediated SIRT7 ubiquitination [394]. Interestingly, inhibition of miR-21-5p led to a decrease in SIRT7 ubiquitination, the LC3II/I ratio, the expression of Beclin1, and an increase in p62 levels. These molecular changes collectively contribute to improved sensitivity to sorafenib [394].

#### 7.2.3. miRNAs Improving Lenvatinib Response

Several miRNAs have been identified as regulators of lenvatinib sensitivity in HCC. miR-128-3p/c-Met axis is involved in the mechanisms underlying lenvatinib resistance by regulating Akt and ERK, which are involved in cell cycle progression and apoptosis, respectively [113]. Wang et al. reported that miR-24-3p suppresses the expression of the anti-apoptotic protein BCL2L2, thereby improving HCC cell sensitivity to lenvatinib treatment [395]. Additionally, miR-34a inhibits autophagy by targeting Beclin-1 in HCC cells, which in turn enhances lenvatinib sensitivity [396].

#### 7.2.4. miRNAs Inducing Lenvatinib Resistance

Several miRNAs have been identified as key players in lenvatinib resistance in HCC. miR-183-5p.1 has been shown to be upregulated in liver tumor-initiating cells (T-ICs), and the overexpression of miR-183-5p.1 promotes self-renewal and tumorigenesis by targeting the MUC15/c-MET/PI3K/Akt/SOX2 axis, thereby reducing lenvatinib sensitivity [397]. Another miRNA implicated in reducing lenvatinib efficacy is miR-520c-3p via its targeting of MBD2, leading to increased FGFR4 expression [398].

Additionally, miR-3154 functions as an oncomiR, is elevated in both HCC and liver cancer stem cells, and plays a significant role in HCC progression by targeting HNF4α and promoting self-renewal, proliferation, metastasis, and tumorigenesis [399].Increased miR-3154 expression was observed in lenvatinib-resistant HCC cell lines, and lenvatinib sensitivity was improved following miR-3154 knockdown. Therefore, miR-3154 may serve as a predictive marker for HCC patient response to lenvatinib treatment, as confirmed through cohort and xenograft analyses, thereby providing valuable insights into the potential clinical benefits of targeting miR-3154 in combination with lenvatinib [399]. Table 7 presents an overview of miRNAs that affect the treatment efficacy of sorafenib or lenvatinib in HCC cells.

## 8. miRNA-Based Therapeutics for Hepatocellular Carcinoma Therapy

**Figure 7:**
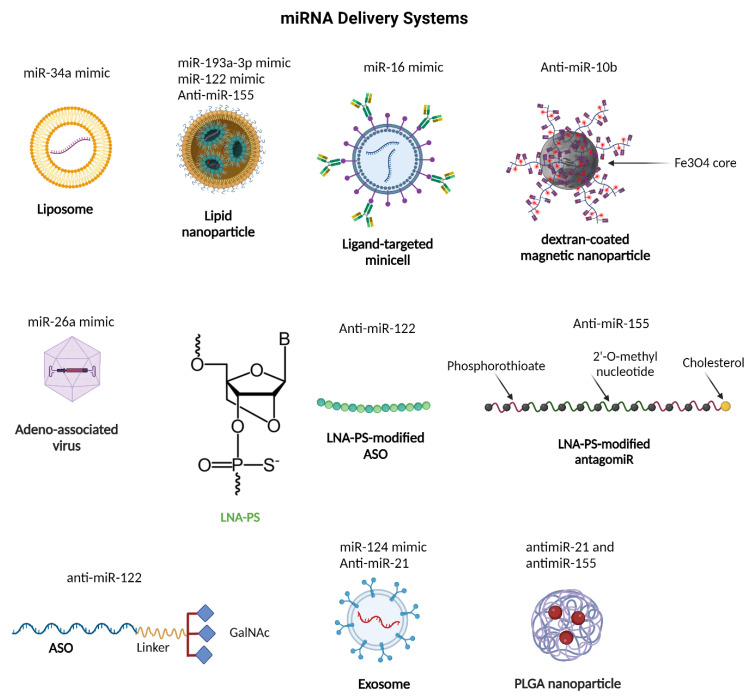
Various tested microRNA delivery systems include liposomes, lipid nanoparticles, ligand-targeted micelles, magnetic nanoparticles, adeno-associated viruses, and exosomes for efficient and targeted delivery to specific tissues. Abbreviation: LNA-PS—Locked Nucleic Acid-Phosphorothioate; ASO—Antisense Oligonucleotide; GalNAc—N-Acetylgalactosamine; PLGA—Poly(lactic-co-glycolic acid). This figure was sourced from BioRender.

miRNA-based therapeutic approaches for HCC treatment offer a dual-pronged strategy: miRNA replacement therapy [419], which introduces tumor-suppressive miRNAs to restore normal function and inhibit tumor growth, and miRNA antagonism [420], which inhibits oncogenic miRNAs to mitigate their adverse effects. These miRNA-targeted strategies are promising because of their ability to simultaneously regulate multiple genes and pathways that are often dysregulated in HCC, offering a more comprehensive approach to cancer therapy than drugs targeting single molecules [421]. To improve in vivo delivery of miRNA-based therapeutics, various strategies can be employed to enhance their stability, targeting efficiency, and cellular uptake while avoiding immunogenic responses. Some promising approaches include nanoparticle-based delivery systems, exosome-mediated delivery, chemical modification, and local delivery (Figure 7) [422].

### 8.1. Restoring Tumor Suppressive miRNA Function

Numerous strategies have been explored in the pursuit of achieving the goal of restoring tumor repressor miRNAs back to the tumor to reduce tumorigenesis. One such approach involves utilizing synthetic miRNA mimics, which are chemically modified versions of specific miRNAs, and these synthetic molecules can be effectively delivered to cancer cells to replicate the actions of tumor-suppressive miRNAs [422]. For instance, miR-34a, which is downregulated in various cancer cells [423], has been the focus of extensive research. Notably, researchers have developed a liposome-formulated miR-34a mimic called MRX34, which has undergone clinical trials in HCC patients with advanced solid tumors [424]. Although trials were terminated owing to the occurrence of five immune-related serious adverse events involving the death of patients [425], the development of MRX34 demonstrated a feasible approach for miRNA drug discovery using liposomal nanoparticles.

In 2014, TargomiRs were introduced to the field of RNA therapeutics as non-living bacterial nanoparticles that serve as effective drug delivery vehicles in which various molecules, including nucleic acids, can be encapsulated [426]. EGFR-targeted TargomiRs loaded with miR-16 mimics have undergone phase I clinical trials in patients with recurrent malignant pleural mesothelioma and non-small cell lung cancer [86]. To avoid undesirable off-target effects, researchers opted to target EGFR using panitumumab because of its high expression in mesothelioma cells. Encouragingly, a phase I study yielded promising results with no reported adverse effects and has paved the way for a phase II clinical trial [86].

Another promising miRNA of interest is miR-193a-3p, which is known to suppress the growth of various cancer types including HCC [427]. To harness this potential, researchers have developed INT-1B3, a lipid nanoparticle-formulated miR-193a-3p mimic [428]. INT-1B3 upregulates the tumor-suppressive PTEN pathway and downregulates several oncogenic pathways in cancer cells [429]. Preclinical studies have demonstrated the safe and effective delivery of INT-1B3 to tumors in vivo [428]. Currently, INT-1B3 is undergoing a phase 1 clinical trial to evaluate its safety, tolerability, pharmacokinetics, pharmacodynamics, and antitumor activity in patients with various solid cancers [421].

Moreover, miR-122, which is frequently downregulated in HCC, has been extensively studied for its tumor suppressive properties. Multiple studies have demonstrated that restoring miR-122 expression inhibits tumor growth, angiogenesis, and metastasis in HCC models [430,431,432]. Hsu et al. successfully delivered miR-122 to HCC cells using cationic lipid nanoparticles consisting of 2-dioleyloxy-N,N-dimethyl-3-aminopropane (DODMA), egg phosphatidylcholine, cholesterol, and cholesterol-polyethylene glycol (LNP-DP1) [433]. This delivery method significantly reduced the expression of miR-122 target genes.

Furthermore, miR-26a plays a crucial role in liver cancer development. Although it is highly expressed in the adult liver, its expression is significantly decreased in human and mouse liver tumors. When miR-26a is delivered to tumors in animal models of liver cancer using adeno-associated virus (AAV), it suppresses cancer cell growth and promotes apoptosis in vivo, resulting in a significant reduction in tumor growth [434].

### 8.2. Inhibition of Oncogenic miRNA Function

Numerous studies have highlighted the potential use of anti-miRs in cancer treatment. One specific example is an LNA-based antagomiR designed to target miR-155, namely Cobomarsen (MRG-106), which has been used in clinical trials in patients with various types of lymphoma e.g., mycosis fungoides, cutaneous T-cell lymphoma, diffuse large B-cell lymphoma, and adult T-cell leukemia/lymphoma. The initial phase I trial demonstrated favorable tolerability and efficacy [435]; however, the subsequent phase II trial was prematurely terminated owing to factors associated with business operations and participant considerations [436]. In another study, nanoparticles were used to simultaneously target miR-21 and miR-155 in lymphoma cell lines. They developed polylactic-co-glycolic acid (PLGA) nanoparticles capable of delivering different classes of anti-miRs, including phosphorothioates (PS) and peptide nucleic acids (PNAs). This innovative approach resulted in significant reductions in the levels of miR-21 and miR-155 and their downstream target genes, and a decrease in cancer cell viability, indicating promising therapeutic potential [437].

A study conducted by Lee et al. focused on the development of lactosylated gramicidin-containing lipid nanoparticles (Lac-GLN) for targeted delivery of anti-miR-155 to HCC cells, resulting in increased expression of miR-155 target genes, such as C/EBPβ and FOXP3. This formulation also exhibited preferential accumulation in mouse hepatocytes [438]. Liang et al. devised a nanoplatform called PCACP (PEI-βCD@Ad-CDM-PEG) to facilitate the delivery of miRNAs. This platform was used to create a miRNA cocktail therapy by encapsulating miR-199a/b-3p mimics and antimiR-10b within PCACP, specifically targeting HCC. This therapy effectively inhibits the proliferation of HCC cells and suppresses tumor growth by targeting specific pathways involved in cancer progression, including mTOR, PAK4, RHOC, and EMT. Notably, in patient-derived xenografts (PDXs), the personalized PCACP/miR-cocktail system showed significant tumor suppression and multi-target regulation, indicating potential improvements over conventional therapies [439].

A promising miRNA inhibitor for the treatment of pancreatic cancer is TTX-MC138, which is specifically designed to suppress the overexpression of miR-10b, an oncogenic miRNA [440]. TTX-MC138 utilizes advanced dextran-coated iron-oxide nanoparticles for enhanced stability and targeted delivery to cancer cells. Preclinical studies have yielded promising results, and further investigation is currently underway [421].

Another avenue of research involves the use of exosomes for the targeted delivery of miRNA-based therapies. In a particular study, researchers explored the co-delivery of 5-FU and an miR-21 inhibitor to colorectal cancer cells using exosomes, where there was effective downregulation of miR-21 expression, resulting in cell cycle arrest, reduced proliferation, increased apoptosis, restoration of tumor suppressor gene expression, and reversal of drug resistance [441].

In addition to targeting oncogenic miRNAs, scientists have developed inhibitors, such as miravirsen and RG101, to combat viral infections. These inhibitors disrupt the activity of miR-122, a liver-specific miRNA that is crucial for hepatitis C virus (HCV) replication [442]. Miravirsen, a 15-mer LNA-PS-modified antisense oligonucleotide (ASO), has been used in clinical trials as a targeted therapy for HCV infections [443]. Similarly, RG101, an N-acetylgalactosamine (GalNAc)-conjugated anti-miR-122 oligonucleotide [444], has shown promise in reducing the viral load in patients with chronic HCV and is currently being tested in a Phase II clinical trial [445]. These inhibitors are potential treatment options for patients that do not respond to traditional therapies. Table 8 presents a compilation of miRNAs that have been studied for targeted delivery in both in vivo experiments and clinical trials, highlighting the diversity of the ongoing research in this field.

## 9. Conclusions and Future Perspectives

HCC represents a significant global health burden owing to its high prevalence and recurrence rates, despite the availability of current therapies. Recent advancements in miRNA sequencing have greatly expanded our understanding of the involvement of miRNAs in hepatocarcinogenesis and drug response. These non-coding RNAs play a crucial role in regulating target genes and signaling pathways, acting as either tumor suppressors or oncogenes, thereby influencing various cancer hallmark traits. Harnessing the potential of miRNAs in HCC therapy offers an innovative approach for molecular cancer treatment. Moreover, serum exosomal miRNAs have emerged as potential biomarkers for monitoring treatment efficacy and improving patient outcome [449]. However, translating miRNA research into clinical application has several critical barriers that need to be addressed. One major challenge is achieving precise modulation of miRNAs in vivo, considering the complex dosage requirements and potential for unforeseen side effects owing to the multitude of gene targets regulated by miRNAs. Additionally, ensuring the stability of miRNA molecules in the bloodstream and their targeted delivery specifically to cancer cells while avoiding adverse effects on normal cells adds further complexity to therapeutic development. To overcome these obstacles, ongoing research has focused on the development of advanced delivery systems such as lipid-based nanoparticles or molecular conjugates to improve the stability and specificity of miRNA-based therapies. Furthermore, identification of key miRNAs that play pivotal roles in liver cancer could refine therapeutic targets, enhance treatment efficacy, and minimize potential side effects.

## Figures and Tables

**Table 1:** miRNAs regulating hepatocellular carcinoma cell survival.

miRNA	Expression in Liver	Target Genes	Pathway	Cellular Process	Refs.
Tumor suppressor miRNAs					
miR-204-5p	Down	RGS20	N/A	Proliferation (−), Apoptosis (+), Cell cycle arrest (G0/G1)	[101]
miR-497-5p	Down	ANXA11	N/A	Proliferation (−), Apoptosis (+), Cell cycle arrest (G0/G1)	[102]
miR-377-3p	Down	RNF38 CPT1C	CPT1C-mediated fatty acid oxidation	Proliferation (−), Apoptosis (+)	[103,104]
miR-559	Down	PARD3 GP73	N/A	Proliferation (−), Autophagy (−)	[105,106]
miR-638	Down	EZH2	N/A	Proliferation (−), Apoptosis (+), Autophagy (+)	[107]
miR-199a-3p	Down	mTOR, c-Met upregulation of ZHX1 and PUMA	mTOR pathway, ZHX1/PUMA signaling	Proliferation (−), Apoptosis (+), Cell cycle arrest (G0/G1)	[108,109]
miR-27a-3p	Down	N/A	PI3K/Akt signaling	Proliferation (−), Apoptosis (+), Cell cycle arrest (G0/G1)	[110]
miR-9	Down	HMGA2	N/A	Proliferation (−), Cell cycle arrest (G0/G1)	[32]
miR-185	Down	RHEB, RICTOR, and AKT1	AKT1 pathway	Cell cycle arrest (G0/G1), Apoptosis (+), Autophagy (+)	[94]
miR-424-5p	Down	E2F7	N/A	Proliferation (−), Cell cycle arrest (G0/G1)	[33]
miR-193a-5p	Down	NUSAP1	N/A	Proliferation (−), Cell cycle arrest (G0/G1), Apoptosis (+)	[42]
miR-621	Down	CAPRIN1	CAPRIN1/CCND2/c-MYC axis	Proliferation (−), Cell cycle arrest (G0/G1)	[34]
miR-125b-5p	Down	KIAA1522	cyclinD1, CDK6, cyclin E and CDK2, and p21	Proliferation (−), Cell cycle arrest (G0/G1), Apoptosis (+)	[35]
miR-29 family (miR-29a, b, c)	Down	RPS15A	cyclin A and cyclin D1, p21	Proliferation (−), Cell cycle arrest (G0/G1), Apoptosis (+)	[37]
miR-450b-3p	Down	PGK1	AKT signaling	Proliferation (−), Cell cycle arrest (G0/G1)	[38]
miR-495	Down	CTRP3	N/A	Proliferation (−), Cell cycle arrest (G0/G1)	[111]
miR-23b-5p	Down	FOXM1	c-MYC and cyclin D1 axis	Proliferation (−), Cell cycle arrest (G0/G1)	[112]
miR-128-3p	Down	c-Met	c-Met signaling	Proliferation (−), Cell cycle arrest (G0/G1), Apoptosis (+)	[113]
miR-875-5p	Down	AEG-1	N/A	Proliferation (−), Cell cycle arrest (G1/S)	[114]
miR-0308-3p	Down	CDK6/Cyclin D1	N/A	Proliferation (−), Cell cycle arrest (G1/S)	[45]
miR-378	Down	Cyclin D1, Bcl-2, Akt, β-catenin and Survivin	N/A	Proliferation (−), Cell cycle arrest (G1/S), Apoptosis (+)	[59]
miR-874	Down	DOR	DOR/EGFR/ERK pathway	Proliferation (−), Cell cycle arrest (G1/S)	[115]
miR-30b-5p	Down	DNMT3A, USP37	N/A	Proliferation (−), Cell cycle arrest (G1/S)	[116]
miR-214-3p	Down	MELK	N/A	Proliferation (−), Cell cycle arrest (G1/S), Apoptosis (+)	[44]
miR-214	Down	Wnt3a	Wnt/β-catenin pathway	Proliferation (−), Cell cycle arrest (G1/S)	[43]
miR-214-5p	Down	E2F2	NF-κB pathway	Proliferation (−), Apoptosis (+)	[117]
miR-409-3p	Down	RAB10	NF-κB pathway	Proliferation (−), Apoptosis (+)	[117]
miR-98-5p	Down	IGF2BP1	N/A	Proliferation (−), Cell cycle arrest (G1/S), Apoptosis (+)	[118]
miR-217	Down	MTDH, EZH2, cyclin-D1, KLF5	N/A	Proliferation (−), Cell cycle arrest (G1/S), Apoptosis (+)	[46,47,48]
miR-302a/d	Down	E2F7	AKT/βcatenin/CCND1 pathway AKT1-cyclin D1 pathway	Proliferation (−), Cell cycle arrest (G1/S), Apoptosis (+)	[119]
miR-1914	Down	GPR39	PI3K/AKT/mTOR pathway	Proliferation (−), Cell cycle arrest (G1/S), Apoptosis (+)	[120]
miR-206	Down	cMET, CCND1, and CDK6	cMet signaling	Proliferation (−), Cell cycle arrest (G1/S), Apoptosis (+)	[65]
miR-22-3p	Down	AKT2	AKT/PI3K pathway	Proliferation (−), Cell cycle arrest (G1/S), Apoptosis (+)	[64]
miR-145-5p	Down	SPATS2, p21 and p27	N/A	Proliferation (−), Cell cycle arrest (G1/S), Apoptosis (+)	[121]
miR-203	Down	MAT2A, MAT2B, NRas	AKT/PI3K pathway RAS/MAPK signaling	Proliferation (−), Cell cycle arrest (S/G2), Apoptosis (+)	[122]
let-7b-5p	Down	p21, CDC25B, HMGA2	CDC25B/CDK1 axis	Proliferation (−) Cell cycle arrest (G2/M)	[49,123]
miR-3613-3p	Down	BIRC5, CDK1, NUF2, ZWINT, and SPC24	N/A	Proliferation (−) Cell cycle arrest (G2/M)	[51]
miR-31-5p	Down	SP1	SP1/cyclin D1	Proliferation (−) Cell cycle arrest (G2/M)	[50]
miR-217	Down	HMGA2	N/A	Proliferation (−) Cell cycle arrest (G2/M)	[124]
miR-448	Down	BCL-2	N/A	Proliferation (−), Apoptosis (+)	[61]
miR-223	Down	NLRP3, Rab1	NLRP3 inflammasome pathway, mTOR pathway	Proliferation (−), Apoptosis (+)	[125]
miR-133b	Down	EGFR, SF3B4	EGFR/PI3K/Akt/mTOR pathway SF3B4/KLF4/KIP1/SNAI2 axis	Proliferation (−) Apoptosis (+)	[62,126]
miR-181a-5p	Down	ATG7	N/A	Proliferation (−), Autophagy (−)	[95]
miR-7	Down	mTOR, ATG5	Autophagy	Proliferation (−), Autophagy (+)	[98,127]
miR-192-3p	Down	XIAP	NF-κB signaling	Apoptosis (+), Autophagy (−)	[128]
miR-519d	Down	Rab10	AMPK Signaling Pathway	Apoptosis (+), Autophagy (+)	[93]
miR-219-5p	Down	NEK6	β-catenin/c-Myc pathway	Proliferation (−)	[129]
miR-375	Down	ErbB2	N/A	Proliferation (−) Apoptosis (+)	[130]
miR-122	Down	miR-21	miR-21–PDCD4 pathway	Proliferation (−) Apoptosis (+)	[131]
miR-4651	Down	FOXP4	N/A	Proliferation (−) Apoptosis (+)	[132]
Let-7b	Down	IGF-1R	N/A	Proliferation (−) Apoptosis (+)	[133]
miR-26a	Down	JAK1	JAK1-STAT3 signaling	Proliferation (−) Apoptosis (+)	[67]
miR-26a/b	Down	ULK1	N/A	Apoptosis (+), Autophagy (−)	[100]
miR-342-3p	Down	MCT1	N/A	Proliferation (−) Apoptosis (+), Necrosis (+)	[134]
miR-654-5p	Down	HSPB1	N/A	Proliferation (−), Ferroptosis (+)	[135]
miR-188-3p	Down	GPX4	N/A	Proliferation (−), Ferroptosis (+)	[136]
miR-612	Down	CoQ10	HADHA-mediated MVA pathway	Proliferation (−), Ferroptosis (+)	[137]
Oncogenic miRNAs					
miR-183-5p	Up	PDCD4	N/A	Proliferation (+), Apoptosis (−)	[138]
miR-494	Up	p27, PUMA, and PTEN	mTOR pathway	Proliferation (+), Cell cycle (induces G1/S transition), Apoptosis (−)	[52]
miR-3682-3p	Up	PHLDA1	PHLDA1-Fas pathway	Proliferation (+), Cell cycle (induces G1/S transition), Apoptosis (−)	[54]
miR-191	Up	KLF6	N/A	Proliferation (+), Cell cycle (induces G1/S transition)	[53]
miR-10b	Up	CSMD1	N/A	Proliferation (+), Cell cycle (induces G1/S transition), Apoptosis (−)	[55]
miR-221-3p	Up	MGMT	N/A	Proliferation (+), Cell cycle (induces G2/M transition), Apoptosis (−)	[56]
miR-21-5p	Up	MELK	AKT/mTOR signaling	Proliferation (+), Ferroptosis (−)	[139]
miR-338-5p	Up	RTN4	N/A	Proliferation (+), Apoptosis (−)	[140]
miR-9-5p	Up	Klf4	AKT/mTOR signaling	Proliferation (+), Apoptosis (−)	[141]
miR-302d	Up	TGFBR2	TGF-β signaling	Proliferation (+), Apoptosis (−)	[142]
miR-371-5p, miR-373 and miR-543	Up	Casp-8	N/A	Necrosis (+)	[85]
miR-106b	Up	DR4	TRAIL pathway	Proliferation (+), Apoptosis (−)	[81]
miR-3174	Up	FOXO1	N/A	Proliferation (+), Apoptosis (−)	[143]
miR-33a	Up	PPARα	N/A	Proliferation (+), Apoptosis (−)	[75]
miR-155	Up	TLR3	TLR3-NF-kB pathway	Proliferation (+), Apoptosis (−)	[80]
miR-93-5p	Up	ERBB4	TETs dependent DNA demethylation	Proliferation (+), Apoptosis (−)	[144]

Note: (−) inhibiting effect; (+) inducing effect.

**Table 2:** miRNAs regulating stemness of hepatocellular carcinoma cells.

miRNA	Target Genes	Pathway	Stemness	Ref.
Tumor suppressor miRNAs				
miR-6838-5p	CBX4	ERK signaling	(−)	[154]
miR-148b	NRP1	ACVR1-BMP-Wnt axis	(−)	[157]
miR-148a	ACVR1	Brg1/Sall4 axis	(−)	[156]
miR-296-5p	Brg1	AKT/βcatenin/CCND1 signaling	(−)	[158]
miR-302a/d	E2F7	N/A	(−)	[119]
miR-589-5p	MAP3K8	SOX9 signaling	(−)	[155]
miR-125a/b	CD90	N/A	(−)	[163]
miR-613	SOX9	PI3K/AKT/mTOR pathway	(−)	[164]
miR-100 and miR-125b	IGF2	hexosamine pathway	(−)	[165]
miR-325-3p	DPAGT1	N/A	(−)	[166]
miR-217	HMGA2	N/A	(−)	[124]
miR-448	MAGEA	AMPK signaling	(−)	[167]
Oncogenic miRNAs				
miR-106b-5p	PTEN	PTEN/PI3K/AKT pathway	(+)	[162]
miR-1246	AXIN2, GSK3β	Wnt/β-catenin pathway	(+)	[160]
miR-5188	FOXO1	β-catenin/Wnt signaling	(+)	[161]
miR-454-3p	CPEB1	N/A	(+)	[168]

Note: (−) inhibiting effect; (+) inducing effect.

**Table 3:** miRNAs regulating hepatocellular carcinoma metastasis.

miRNA	Target Genes	Pathway	Cellular Process	Refs.
Anti-metastatic miRNAs				
miR-320a	HMGB1, c-Myc	N/A	N/A	[202,203]
miR-29c-3p	ADAM12	N/A	N/A	[204]
miR-188-5p	FOXN2	N/A	N/A	[205]
miR-142-3p	ZEB1, TGF-β1, HMGB1	TGF-β pathway	EMT, Angiogenesis	[206,207,208]
Exosomal miR-125b	SMAD2	TGFβ1/SMAD signaling	EMT	[209]
Exosomal miR-29b	DNMT3b	N/A	EMT	[210]
miR-10a-5p	SKA1	N/A	EMT	[211]
miR-2113	WDR66	N/A	EMT	[195]
miR-30e	MTA1	MTA1/ErbB2 axis	EMT	[212]
miR-517c	KPNA2	PI3K/AKT pathway	EMT	[213]
miR-130a-3p	AR	N/A	EMT, Angiogenesis	[176]
miR-526b-5p	HGF	HGF/c-Met pathway	EMT, Angiogenesis	[214]
miR-4270-5p	SATB2	N/A	EMT	[215]
miR-124-3p	CRKL, Sp1	RAF/MEK/ERK1/2 pathways	EMT	[177,216]
miR-875-5p	AEG1/MTDH	N/A	EMT	[114]
miR-296-5p	NRG1	NRG1/ERBB2/ERBB3/RAS/MAPK/Fra-2 signaling	EMT	[159]
miR-449a	Notch1	Notch pathway	EMT	[217]
miR-30a	Beclin 1, Atg5	Autophagy pathway	Autophagy	[99]
miR-7	FAK	Akt pathway	N/A	[190]
miR-148a-3p	SMAD2	TGF-β signaling	N/A	[218]
miR-298	CTNND1	Wnt/β-catenin signaling	N/A	[219]
miR-409-3p	BRF2	Wnt/β-catenin signaling	N/A	[220]
miR-466	MTDH/AEG-1	N/A	N/A	[221]
miR-138-5p	FOXC1	N/A	N/A	[222]
miR-455-5p	IGF-1R	IGF-1R-AKT-GLUT1 axis	N/A	[223]
miR-139-3p	UCK2	N/A	N/A	[224]
miR--219-5p	NEK6	β-catenin/c-Myc pathway	N/A	[225]
miR-145	SERPINE1	HIF-1 signaling pathway	N/A	[226]
miR-515–5p	IL6	IL-6/JNK/STAT3 signaling	N/A	[227]
miR-378a-3p	PD-L1	STAT3 signaling	Immune response	[228]
miR-199a/b-5p	ROCK1	ROCK1/MLC and PI3K/Akt signaling	N/A	[229]
miR-378a-5p	VEGF	VEGF pathway	Angiogenesis	[230]
miR-495	IGF1R	N/A	N/A	[231]
miR-194-3p	MMP9	N/A	ECM degradation	[232]
miR-489	MMP7	N/A	ECM degradation	[233]
miR-211-5p	ACSL4	N/A	N/A	[175]
miR-342-3p	AGR2	N/A	Apoptosis, cell cycle, ECM degradation	[234]
miR-767-3p	SMIM7	N/A	N/A	[235]
miR-139	GDF10	N/A	N/A	[236]
miR-15a-3p	HMOX1	N/A	N/A	[179]
miR-449 family	SOX4	TGF-β pathway	N/A	[237]
miR-708	SMAD3	TGF-β pathway	N/A	[238]
miR-300	LEF-1	N/A	N/A	[239]
miR-98	EZH2, IL-10	Wnt/β-catenin pathway	N/A	[240,241]
Metastatic miRNAs				
miR-362-3p	CD82	TGF-β signaling	EMT	[242]
miR-625-3p	PDLIM5	N/A	EMT	[243]
miR-92a-3p	PTEN	Akt/Snail pathway, PI3K/AKT/mTOR Signaling	EMT	[244,245]
miR-93-5p	directly upregulates MAP3K2, inhibits ERBB4 and TETs	MAP3K2/ p38-JNK/p21 signaling pathway	N/A	[144,201]
miR-10a-5p	NCOR2	Wnt/β-catenin pathway	EMT	[246]
miR-HCC3	TNFRSF19, RAB43	N/A	EMT	[247]
miR-197	Axin-2, NKD1, DKK2	Wnt/β-catenin pathway	EMT	[248]
miR-21-3p	SMAD7	SMAD7/YAP1 axis	EMT	[249]
miR-3691-5p	PTEN	PI3K/Akt signaling	N/A	[250]
miR-106b	PTEN	N/A	N/A	[251]
miR-18a	KLF4, Bcl2L10	N/A	N/A	[196,197]
miR-106b-5p	FOG2	N/A	N/A	[199]
miR-376c-3p	ARID2	N/A	N/A	[200]
miR-182-5p	FOXO3a	AKT/FOXO3a pathway, Wnt/β-catenin signaling	N/A	[252]
miR-769-5p	RYBP	N/A	N/A	[253]
miR-1251-5p	AKAP12	N/A	N/A	[254]
miR-483-5p	Positive regulator of IGF-II	N/A	N/A	[255]
miR-106b-5p	GPM6A	AKT/ERK signaling	N/A	[256]
Exosomal miR-25-5p	SIK1 Fbxw7, LRRC7	Wnt/β-catenin signaling	N/A	[198,257,258]
Exosomal miR-21	PTEN	PDK1/AKT signalling	N/A	[259]
Exosomal miR-1273f	LHX6	Wnt/β-catenin signaling	EMT	[260]
Exosomal miR-92a-3p	PTEN	Akt/Snail signaling	N/A	[244]
Exosomal miR-224	GNMT	N/A	N/A	[261]
Extracellular vesicle (EV)-miR-3129	TXNIP	N/A	N/A	[262]
Exosomal miR-1247-3p	B4GALT3	β1-integrin/NF-κB signaling	N/A	[263]

**Table 4:** miRNAs regulating tumor microenvironment in hepatocellular carcinoma.

miRNA	Source of Cell Type	Target Gene	Pathway	Function	Ref.
Tumor suppressor miRNAs					
Exosomal miR-320a	CAFs	PBX3	MAPK pathway	Inhibits HCC proliferation and metastasis	[302]
Exosomal miR-150-3p	CAFs	N/A	N/A	Inhibits HCC invasion	[282]
miR-101-3p/miR-490-3p	CAFs	TGFBR1	N/A	Induce infiltration of myeloid-derived suppressor cells, regulatory T cells, and M2 macrophages	[283]
miR-26a	HCC cells	M-CSF	PI3K/Akt pathway	Inhibits macrophage recruitment and M2 polarization	[303]
Exosomal miR-628-5p	M1 macrophages	METTL14	circFUT8/miR-552-3p/CHMP4B pathway	Inhibits HCC progression	[304]
miR-144/miR-451a	HCC cells	HGF, MIF	N/A	Induce macrophage M1 polarization	[288]
Exosomal miR-125a/b	M2 macrophages	CD90	N/A	Inhibit cell proliferation and stem cell properties	[163]
miR-148b	HCC cells	CSF1	CSF1/CSF1R pathway	Inhibits TAM infiltration, HCC growth and metastasis	[289]
miR-28-5p	HCC cells	IL-34	TGFβ1 signaling	Inhibits TAM infiltration, HCC growth and metastasis	[290]
miR-200	HCC cells	PD-L1	N/A	Induces CD8+ T cells viability, inhibits metastasis	[305]
miR-145	HCC cells	PD-L1	PI3K/AKT signaling	Inhibits tumor growth, EMT, and metastasis	[306]
miR-194-5p	HCC cells	PD-L1 and PD-L2	N/A	Reduces cytotoxic T cells apoptosis	[307]
miR-223	HCC cells	HIF1α	CD39/CD73-adenosine pathway	Inhibits infiltration of PD-1+ T cells and PD-L1+ macrophages, inhibits angiogenesis	[293]
miR-329-3p	HCC cells	KDM1A	N/A	Inhibits the expression of PD-L1 in HCC cells via increasing MEF2D methylation, inhibits tumor growth	[294]
miR-200c	HBV+ HCC cells	CD274	HBV-pSTAT3-SALL4-miR-200c-PD-L1 axis	Inhibits HBV-mediated PD-L1 expression and CD8+ T cell exhaustion	[295]
miR-22	HCC cells	HIF1α	Retinoic acid signaling	Inhibits IL17 signaling, expands cytotoxic T cells and reduces Treg	[296]
miR-30-5p	HCC cells	Snail	Snail-DDP4- CXCL10 axis	Induces CD8+ T cell infiltration	[299]
miR-570	HCC cells	CD31 and VEGF	N/A	Increases CD8+IFN-γ+ T cells, induces apoptosis, inhibits angiogenesis	[308]
miR-374b	CIK cells	PD-1	N/A	Induces cytotoxicity of cytokine-induced killer cells	[309]
miR-1258	NK cells and DC cells	N/A	N/A	Stimulates TLR7/8 expression, activates NKs and promotes DCs maturation, inhibits tumor growth and metastasis	[300]
Oncogenic miRNAs					
Exosomal miR-1247-3p	HCC cells	B4GALT3	β1-integrin–NF-κB signaling	Induces CAFs activation and lung metastasis	[263]
Exosomal miR-4508	HCC cells	RFX1	RFX1-IL17A-p38 MAPK-NF-κB pathway	Activates lung fibroblasts and induces lung metastasis	[281]
Exosomal miR-21	HCC cells	PTEN	PDK1/AKT signaling	Converts normal HSCs to CAFs, induces angiogenesis	[259]
Exosomal miR-200b-3p	HCC cells	ZEB1	JAK/STAT signaling	Induces macrophage M2 polarization	[310]
Exosomal miR-4669	HCC cells	N/A	N/A	Induces M2 macrophage polarization, migration ability, and sorafenib resistance	[286]
Exosoma miR-92a-2-5p	M2 macrophages	AR	AR/PHLPP/p-AKT/β-catenin signaling	Induces HCC cells invasion	[311]
Exosomal miR-27a-3p	M2 macrophages	TXNIP	N/A	Induces HCC cells stemness, proliferation, drug resistance, migration, invasion, and tumorigenicity	[312]
Exosomal miR-660-5p	M2 macrophages	KLF3	N/A	Induces EMT	[313]
Exosomal miR-452-5p	HCC cells	TIMP3	N/A	Induces M2 macrophage polarization,	[287]
miR-889	HCC cells	MICB	N/A	Reduces NK cell-mediated cytotoxicity	[301]
miR-561-5p	HCC cells	CX3CL1	STAT3 signaling	Reduces CX3CR1+ NK cell infiltration, induces tumor growth and lung metastasis	[4]

**Table 5:** miRNAs regulating hepatocellular carcinoma angiogenesis.

miRNA	Target Genes	Pathway	Ref.
Anti-angiogenic miRNAs			
miR-26a	HGF	HGF/c-Met pathway	[331]
miR-526b-5p	HGF	HGF/c-Met pathway	[214]
miR-200b	Transcription factor ERG	N/A	[322]
miR-203a	HOXD3	N/A	[318]
miR-144-3p	SGK3	N/A	[320]
miR-375	PDGFC, AEG-1	N/A	[332]
miR-3064-5p	FOXA1	FOXA1/CD24/Src pathway	[323]
miR-199a-3p	MMP2, HGF, VEGFA, VEGFR1	N/A	[316]
miR-378a-5p	VEGF	VEGF pathway	[230]
miR-223	HIF1α	CD39/CD73-adenosine pathway	[293]
miR-1296	E2F7	N/A	[333]
Pro-angiogenic miRNAs			
Let-7i-5p	TSP1	N/A	[334]
miR-210-3p	SMAD4, STAT6	N/A	[330]
miR-130b-3p	HOXA5	N/A	[328]

**Table 6:** miRNAs regulating chemotherapy resistance in hepatocellular carcinoma.

miRNA	Target Genes	Drug Response	Cellular Process	Pathway	Ref.
miRNAs improving drug sensitivity					
miR-375	MDR1, AEG1, YAP1, and ATG7	doxorubicin	Proliferation, autophagy	N/A	[349]
miR-223	ABCB1	doxorubicin	N/A	N/A	[350]
miR-125b	HIF1A, YBX1, PDGFRB	doxorubicin	stemness	HIF1α/PDGFβ/pAKT	[338]
miR-383	EIF5A2	doxorubicin	Proliferation, apoptosis	N/A	[351]
miR-140-5p	PIN1	doxorubicin	Proliferation	N/A	[352]
miR-590-5p	YAP1	doxorubicin	Proliferation, stemness	Hippo signaling	[353]
miR-122	ABCB1 and ABCF2	doxorubicin	Cell cycle	N/A	[354]
miR-122	PKM2	doxorubicin	Glycolysis	N/A	[355]
miR-760	Notch1	doxorubicin	Proliferation, apoptosis	Notch1/Hes1-PTEN/Akt Signaling	[356]
miR-26a/b	ULK1	doxorubicin	Autophagy, apoptosis	N/A	[100]
miR-218-5p	EIF5A2	doxorubicin	N/A	N/A	[357]
miR-325-3p	DPAGT1	doxorubicin	N/A	Hexosamine pathway	[166]
miR-223	FOXO3a	doxorubicin	Autophagy	N/A	[339]
miR 361 5p	MAP3K9	cisplatin	Apoptosis	N/A	[341]
miR-610	HDGF	cisplatin	Proliferation and apoptosis	N/A	[342]
miR-10a-5p	MSI1	cisplatin	Proliferation and apoptosis	AKT signaling	[344]
miR-27a-3p	ABCB1	5-fluorouracil cisplatin	Proliferation and apoptosis	PI3K/Akt pathway	[110,358]
miR-203a	BMI1	5-fluorouracil	Proliferation, stemness	N/A	[359]
miR-125b	HK II	5-fluorouracil	Glycolysis	N/A	[360]
miR-216b	MALAT1	5-fluorouracil	Autophagy	N/A	[340]
miR-145	TLR4	5-fluorouracil	Apoptosis	N/A	[361]
miR-138-5p	HIF-1α	Radiosensitivity	N/A	Migration/invasion, EMT	[362]
miRNAs inducing drug resistance					
miR-182	TP53INP1	cisplatin	Viability	N/A	[348]
miR-551a	MEF2C	5-fluorouracil	Viability and sphere formation	N/A	[347]
Exosomal miR-32-5p	PTEN	5-fluorouracil	Angiogenesis, EMT	PI3K/Akt pathway	[363]
miR-24 and miR-221	caspase 8/3	TRAIL	Angiogenesis	N/A	[364]

**Table 7:** miRNAs regulating targeted therapy resistance in hepatocellular carcinoma.

miRNA	Target Genes	Targeted Therapy Agent	Cellular Process	Pathway	Refs.
miRNAs improving drug sensitivity					
Exosomal miR-744	PAX2	Sorafenib	N/A	N/A	[400]
miR-3689a-3p	CCS	Sorafenib	mitochondrial oxidative stress, Apoptosis	CCS/SOD1 axis	[389]
miR-124	CAV1	Sorafenib	Stemness	N/A	[401]
miR-338-3p	RAB1B	Sorafenib	Apoptosis, invasion	N/A	[402]
miR-122	IGF-1R	Sorafenib	Apoptosis	RAS/RAF/ERK signaling	[403]
miR-345-5p	TOP2A	Sorafenib	Apoptosis	N/A	[388]
miR-122	SerpinB3	Sorafenib	Apoptosis	N/A	[404]
miR-138-1-3p	PAK5	Sorafenib	Apoptosis	β-catenin/ABCB1 signaling	[386]
miR-654-5p	HSPB1	Sorafenib	Ferroptosis	N/A	[135]
miR-182-5p	N/A	Sorafenib	EMT	N/A	[405]
miR-128	CD151	Sorafenib	EMT	N/A	[406]
miR-1294	TGFβR1	Sorafenib	EMT	N/A	[407]
miR-16-5p	cyclin E1	Sorafenib	Cell cycle	PTEN/Akt signaling	[408]
miR-449a-5p	PEA15, PPP1CA, TUFT1	Sorafenib	N/A	AKT and ERK signaling	[372]
miR-4277	CYP3A4	Sorafenib	Drug metabolism	N/A	[373]
miR-374b	hnRNPA1	Sorafenib	Aerobic glycolysis	PKM2	[409]
miR-30a-5p	ATG5, Beclin-1, CLCF1	Sorafenib	Autophagy, aerobic glycolysis	PI3K/AKT signaling	[375,376]
miR-204	ATG3	Sorafenib	Autophagy	N/A	[377]
miR-378a-3p	IGF1R	Sorafenib	N/A	ERK/PI3K signaling	[380]
miR-140-3p	PXR	Sorafenib	Drug clearance	N/A	[383]
miR-375	PDGFC, AEG-1, ATG14	Sorafenib	Angiogenesis, autophagy	N/A	[332,410]
miR-124-3p.1	AKT2, SIRT1	Sorafenib	N/A	FOXO3a pathway	[385]
miR-142-3p	ATG5, ATG16L1	Sorafenib	Autophagy	N/A	[378]
miR-148a	PXR	Sorafenib	Drug clearance	N/A	[384]
miR-486-3p	FGFR4, EGFR	Sorafenib	N/A	N/A	[411]
miR-3163	ADAM-17	Sorafenib	N/A	Notch signaling	[412]
miR-483-5p	PPARα, TIMP2	Sorafenib	Apoptosis, steatosis, fibrosis	Notch signaling	[413]
miR-3163	ADAM17	Sorafenib	N/A	Notch signaling	[412]
miR-5590-3p	PINK1	Lenvatinib	Apoptosis	N/A	[414]
miR-128-3p	c-Met	Lenvatinib	Apoptosis, cell cycle	c-Met pathway	[113]
miR-24-3p	BCL2L2	Lenvatinib	Apoptosis	N/A	[395]
miR-34a	Beclin-1	Lenvatinib	Autophagy	N/A	[396]
miRNAs inducing drug resistance					
miR-21-5p	USP24	Sorafenib	Autophagy	USP24-SIRT7 axis	[394]
miR-23a-3p	ACSL4	Sorafenib	Ferroptosis	N/A	[390]
miR-125b-5p	Ataxin-1	Sorafenib	EMT	N/A	[392]
miR-494	p27, PUMA, PTEN, G6pc	Sorafenib	Cell cycle, Survival, invasion, stemness, glycogenolysis, gluconeogenesis	mTOR pathway	[52]
miR-126-3p	SPRED1	Sorafenib	N/A	ERK signaling pathway	[415]
miR-96	TP53INP1	Sorafenib	promotes liver T-ICs expansion	N/A	[416]
miR-3677-3p	FBXO31	Sorafenib	Proliferation, invasion, stemness, apoptosis	N/A	[417]
miR-4669	SIRT1	Sorafenib	Immunosuppressive TME	N/A	[286]
miR-223	FBW7	Sorafenib	N/A	N/A	[418]
miR-183-5p.1	MUC15	Lenvatinib	Proliferation, apoptosis, stemness	c-MET/PI3K/AKT/SOX2 signaling	[397]
miR-520c-3p	MBD2	Lenvatinib	Proliferation, cell cycle	GF19/FGFR4/FRS2 signaling	[398]
miR-3154	HNF4α	Lenvatinib	Proliferation, apoptosis, stemness	N/A	[399]

**Table 8:** miRNAs as therapeutics for cancer treatment.

Therapeutic Molecule	Target miRNA	Disease	Phase	Delivery Platform	Route of Administration	Ref.
miRNA replacement therapy						
MRX34	miR-34a	Advanced solid tumors	Phase I	Liposomal	IV	[446]
TargomiR	miR-16	MPM, NSCLC	Phase I	EGFR targeting minicell	IV	[86]
INT-1B3	miR-193a-3p	Advanced solid tumors	Phase I	LNP	IV	[428]
N/A	miR-124	Pancreatic cancer	Preclinical	Exosome	SC	[447]
N/A	miR-122	HCC	Preclinical	LNP	IV	[433]
N/A	miR-26a	HCC	Preclinical	MSCV-derived retroviral construct	IV	[434]
N/A	miR-22	HCC	Preclinical	AAV	IV	[402]
miRNA inhibition therapy						
MRG-106 (cobomarsen)	miR-155	CTCL, CLL, DLBCL, ATLL	Phase II	LNA	IT, SC, IV	[436]
Miravirsen	miR-122	Chronic HCV	Phase II	LNA-PS-modified ASO	SC	[443]
RG-101	miR-122	Chronic HCV	Phase II	GalNAc-ASO	SC	[445]
TTX-MC138	miR-10b	PAC	Preclinical	NP	IV	[448]
N/A	miR-155	HCC	Preclinical	LNP	IV	[438]
N/A	miR-21	CRC	Preclinical	Exosome	IV	[441]
N/A	miR-21 and miR-155	Lymphoma	N/A	NP containing PS and PNA	N/A	[437]
